# Kaempferol Regulates Lipid Homeostasis, Endocannabinoid System, and PPARα in Rat Cerebral Cortex Following BCCAO/R

**DOI:** 10.3390/biom15101440

**Published:** 2025-10-11

**Authors:** Gianfranca Carta, Maria Pina Serra, Elisabetta Murru, Marianna Boi, Claudia Manca, Ylenia Lai, Monica Cabboi, Antonella Carta, Sebastiano Banni, Marina Quartu

**Affiliations:** Department of Biomedical Sciences, University of Cagliari, 09100 Cagliari, Italy

**Keywords:** bilateral-common carotid artery occlusion, cerebral hypoperfusion/reperfusion, neuroinflammation, endocannabinoids, *N*-acylethanolamines, cannabinoid receptors, PPARα, COX-2, GFAP, Iba1

## Abstract

Previous research has demonstrated that the transient bilateral common carotid artery occlusion and reperfusion (BCCAO/R) effectively models early brain inflammation resulting from sudden hypoperfusion and subsequent reperfusion. According to studies showing that diet and nutrition strongly influence brain neuroplasticity, in this study we evaluated whether kaempferol (KAM), a dietary flavonoid, offers neuroprotection in a rat BCCAO/R model. Adult Wistar rats were gavage fed a single dose of KAM (40 mg) six hours before surgery. Comprehensive lipidomic and molecular analyses were conducted on samples from the frontal and temporal-occipital cortices, as well as the plasma. In the frontal cortex, KAM elevated anti-inflammatory *N*-acylethanolamines palmitoylethanolamide (PEA), oleoylethanolamide (OEA), and docosahexaenoylethanolamide (DHAEA) and reduced oxidized arachidonic acid metabolites. KAM also downregulated cyclooxygenase- 2 (COX-2) protein and selectively decreased the endocannabinoid 2-arachidonoylglycerol (2-AG), showing a shift in AA metabolism. These molecular changes correlated with increased levels of peroxisome proliferator-activated receptor alpha (PPARα) and cannabinoid receptors CB1R and CB2R, supporting activation of both nuclear and membrane-bound anti-inflammatory pathways. No significant changes were observed in the temporal-occipital cortex. In plasma, DHAEA levels increased similarly to those in the cortex. However, rises in PEA and OEA were detected only in sham-operated KAM-treated animals, suggesting possible central redistribution under hypoperfusion/reperfusion stress. In summary, these findings demonstrate that KAM exerts dual anti-inflammatory effects by inhibiting COX-2-mediated prostanoid synthesis and promoting PPARα-driven lipid signaling. This dual mechanism highlights the potential of KAM as a dietary intervention to reduce neuroinflammation associated with hypoperfusion–reperfusion challenges.

## 1. Introduction

Clinical and preclinical research suggest that inflammation and oxidative stress play crucial roles in the pathogenesis of cerebral ischemia, providing valuable insights into potential therapeutic targets [1]. The inflammation plays a critical role in amplifying and propagating neuronal damage following hypoperfusion and revascularization injuries [2,3]. Although reperfusion restores the blood flow, the uncontrolled cerebral tissue responses to the sudden upsurge of oxygen availability can lead to maladaptive plasticity and irreversible neuronal damage. Given the unique architecture of the cerebral vascular bed, characterized by limited collateral circulation [4], any loss of blood supply rapidly disrupts the physiological tissue balance. This triggers a series of adaptive tissue responses driven by factors such as the activation of signaling lipid pathways, an imbalance of neurotrophic factors, the upregulation of neuronal receptors, and the recruitment of microglia [5,6,7,8,9,10]. In this context, preventive strategies, particularly those addressing carotid artery stenosis, have gained significant research interest.

The transient bilateral common carotid artery occlusion and reperfusion (BCCAO/R) does not induce a genuine ischemic injury in the rodent brain, as collateral circulation efficiently enables restoration of cerebral blood flow within minutes [11,12,13]. However, the BCCAO/R model is useful for studying acute cerebral oxidative stress and pro-inflammatory responses resulting from sudden hypoperfusion followed by reperfusion [14]. Experimental research shows that this model affects brain tissue homeostasis and is linked to molecular changes, such as increased lipid peroxidation and enhanced activity of superoxide dismutase [15]. It also results in an imbalance of the tissue lipid profile and activation of the endocannabinoid system (ECS), with increases in cyclooxygenase-2 (COX-2) activity [7,8,16,17] detectable within hours of the surgery.

In this context, there is a growing interest in natural compounds that can reduce brain damage caused by reperfusion. Kaempferol (KAM) (3,4,5,7-tetrahydroxyflavone) is a dietary flavonol, naturally found in different plant species and red wine [18,19], often occurring in its glycosylated forms [20]. In plants, the KAM glycosides exhibit a range of phytochemical properties, including regulation of the defense mechanisms, efficiently removing reactive oxygen species, modulating gene expression to induce production of stress-related plant hormones, such as salicylic acid [21], and regulating the cell cycle [22]. Traditional Chinese and Spanish medicines indicate that KAM has anti-inflammatory properties [23]. Other reported benefits include its analgesic properties, anti-aging effects, anti-cancer potential, management of diabetes and related complications, anti-fertility effects, antipyretic effects, modulation of the central nervous system (CNS), liver protection, and wound healing [19,23,24]. Human and early preclinical studies show that KAM displays broad therapeutic properties against various chronic inflammatory conditions [25], diabetes, and cardiovascular disease [23,26]. Additionally, KAM glycosides are linked to neuroprotection by mitigating neuroinflammation, which plays a role in various neurological conditions [23,27,28], including some types of stroke [29]. In the nervous system, KAM glycosides can block the production of key inflammatory signals, such as cytokines and enzymes, including COX-2 [25].

Computer-based studies demonstrate that KAM can stimulate the peroxisome proliferator-activated receptor alpha (PPARα) [30].

PPARα is a ligand-activated transcription factor of the nuclear hormone receptor family [31,32,33]. By regulating gene expression, it controls crucial metabolic processes and inflammatory responses in peripheral organs and the brain [34,35,36], partly through the inhibition of pro-inflammatory molecules [37,38]. PPARα also upregulates genes involved in fatty acid transport and mitochondrial and peroxisomal fatty acid β-oxidation [32,39,40], a pathway essential for long- and medium-chain fatty acid metabolism.

BCCAO/R-induced oxidative stress is associated with specific changes involving fatty acids and signaling lipid molecules such as the *N*-acylethanolamines (NAEs), which are congeners of the endocannabinoids (eCBs) [7]. While eCBs, namely anandamide (AEA) and 2-arachidonoylglycerol (2-AG), both derived from arachidonic acid (AA), primarily act on cannabinoid receptors (CBRs), certain NAEs are known to act as endogenous agonists of PPARα [41,42,43,44]. Our earlier work showed that the in vivo administration of dietary natural compounds, such as *Pistacia lentiscus* L. essential oil [16], the phytocannabinoid β-caryophyllene [9], and the polyphenol Resveratrol [45] may help the brain tissue to cope better with the hypoperfusion/reperfusion metabolic challenge.

In this study, we utilized the BCCAO/R rat brain model of hypoperfusion/reperfusion to investigate whether a single acute dose of KAM could have a neuroprotective effect in the cerebral cortex. To this aim, we examined the forebrain areas directly and selectively vascularized by the internal carotid artery branches, which are most affected by the BCCAO/R [7], and the temporal-occipital cortex, which, being served by the basilar artery branches, can be used as a control. We analyzed the brain tissue and plasma fatty acid profiles, lipoperoxides (molecules involved in neuroinflammation and indicative of oxidative stress), with a focus on AA hydroperoxides (AA_HP), the receptors CB1R, CB2R, PPARα and their endogenous ligands, as well as the enzyme COX-2 before and after the BCCAO/R, with or without KAM. We found that KAM administration was associated with modulation of tissue and plasma fatty acid profiles and reduction in AA_HP in brain regions sensitive to BCCAO/R. KAM also affected the expression of key inflammatory markers, including COX-2, CBR, and PPARα. To evaluate the systemic action of KAM, we also investigated the AA_HP, eCBs, and eCB congeners in plasma. To assess the influence of KAM on brain tissue challenged by the BCCAO/R, we also studied the glial fibrillary acidic protein (GFAP) and ionized calcium-binding adapter molecule 1 (Iba1). The results are discussed in view of the potential significance of these molecules as early indicators of short-term cerebral global blood flow loss and of the prospect of using KAM as a dietary supplement to help the brain tissue cope with the pro-inflammatory response associated with the hypoperfusion/reperfusion challenge.

## 2. Materials and Methods

### 2.1. Animals and Keeping

Adult male Wistar rats (Harlan, Udine, Italy) weighing an average of 210 ± 20 g (mean ± SD) were housed for one week before the experiment under controlled conditions. The temperature was maintained at 21 ± 2 °C, with a 12 h light/dark cycle, and relative humidity was set at 60 ± 5% to minimize any distress to the animals. Rat care and handling adhered to institutional guidelines, following national (D.L. n. 116, Gazzetta Ufficiale della Repubblica Italiana, Additional 40, 18 February 1992, and subsequent modifications) and international laws and policies (EEC Council Directive 86/609, OJ L 358, 1, 12 December 1987). The rats had unrestricted access to standard laboratory food (A04, Safe, Augy, France) and water.

Animals were fasted for 12 h before surgery. A total of 76 rats were included in the study. Rats were randomly assigned to four groups, the sham-operated ones and those subjected to BCCAO/R treated with the vehicle alone or with the vehicle containing the KAM, and named as follows: SHAM-vehicle, BCCAO/R-vehicle, SHAM-KAM, and BCCAO/R-KAM. Six hours before the surgery, the rats were gavage fed a pre-treatment consisting of either the vehicle, i.e., 0.3 mL of sunflower oil, or KAM (Sigma-Aldrich, St. Louis, MO, USA) as a single dose of 40 mg in 0.3 mL vehicle/per rat (corresponding to 180 mg/kg^−1^). The selected dosage was based on our previous research on the neuroprotective effects of other natural substances [9,16,45]. The choice to use a vehicle was made to ensure that an accurate amount of KAM could be administered via gavage and to facilitate its absorption.

### 2.2. Surgery

The surgical procedure, adapted from the method of Iwasaki et al. [46], was performed between 1:00 and 4:30 p.m. Anesthesia was induced via an intraperitoneal injection of Equithesin (16.2% *w*/*w* pentobarbital, 4.2% *w*/*v* chloral hydrate, 39.6% *w*/*w* propylene glycol, 2.12% *w*/*v* MgSO_4_, and 10% *w*/*w* ethanol in sterile distilled water) at a dosage of 0.5 mL per 100 g of body weight, as described in previous studies employing the BCCAO/R model [16]. The solution was administered slowly with a fine-gauge needle into the lower right abdominal quadrant to minimize discomfort and ensure even distribution. No respiratory distress or abdominal distension was observed. Anesthetic depth was monitored and maintained throughout the procedure by assessing pedal withdrawal reflex and respiratory patterns. No supplemental anesthesia was required.

A midline neck incision was performed, and blunt muscle dissection exposed the common carotid arteries (CCAs), with care taken to avoid vagus nerve injury. Cerebral blood flow was reduced by clamping the CCAs for 30 min using two atraumatic microvascular clips. Reperfusion was initiated by removing the clips, restoring blood flow for 60 min. Sham-operated rats underwent the same surgical procedure without CCA occlusion and served as control animals to assess the effects of anesthesia and surgical manipulation.

### 2.3. Sampling

Following BCCAO/R, brain samples were collected as either fresh tissue for lipid analysis (*n* = 28) and Western blot (*n* = 32) or after fixation by transcardial perfusion with ice-cold fixative solution (4% paraformaldehyde in 0.1 M phosphate buffer, pH 7.3) for immunohistochemistry (*n* = 16). For lipid and Western blot analysis, the cerebral cortex was rapidly dissected from the rest of the brain and transversely cut at the approximate bregma level of −1.0 mm [47] for the frontal cortex, and −4.5 mm for the temporal-occipital cortex, which served as a control region not supplied by internal carotid artery branches. Specimens were then frozen at −80 °C until analysis. Blood was collected from the trunk of euthanized animals (*n* = 28) and centrifuged at 1500 g for 10 min at 2–8 °C, and the resulting plasma was stored at −80 °C until lipid assay. For immunohistochemical assays, brains were post-fixed by immersion in freshly prepared fixative (pH 7.3) for 4–6 h at 4 °C and then rinsed in 0.1 M phosphate buffer containing 20% sucrose. Investigators remained blinded to the experimental conditions throughout each assay.

### 2.4. Measurement of Fatty Acid in Brain Tissue and Plasma

Frozen tissues from SHAM-vehicle, BCCAO/R-vehicle, SHAM-KAM, and BCCAO/R-KAM rats (*n* = 7 per group) were homogenized. Total lipids were extracted from specific brain regions and plasma using a chloroform and methanol mixture (2:1, *v*/*v*) containing 2 μg of vitamin E [48]. Aliquots underwent mild saponification as previously described [49] to yield free fatty acids for high-performance liquid chromatography (HPLC) analysis. Unsaturated fatty acids were measured using an Agilent 1100 HPLC system (Agilent Technologies, Santa Clara, CA, USA) with a diode array detector, and saturated fatty acid by Gas Chromatography (GC) (Agilent, Model 6890, Palo Alto, CA, USA) equipped with a flame ionization detector [50].

### 2.5. Endocannabinoid and Congener Quantification in Tissue and Plasma

Frozen tissues from SHAM-vehicle, BCCAO/R-vehicle, SHAM-KAM, and BCCAO/R-KAM rats (n = 7 per group) were homogenized. The eCBs and eCB congeners were extracted from various brain regions and plasma using a chloroform–methanol solution (2:1, *v*/*v*), containing internal deuterated standards for AEA, 2-AG, palmitoylethanolamide (PEA), oleoylethanolamide (OEA), and docosahexaenoylethanolamide (DHAEA). Quantification was performed by isotope dilution with [2H]^8^ AEA, [2H]^5^ 2-AG, [2H]^4^ PEA, and [2H]^4^ OEA (Cayman Chemical, Ann Arbor, MI, USA). The levels of AEA, 2-AG, PEA, OEA, and DHAEA were measured by liquid chromatography–atmospheric pressure chemical ionization–mass spectrometry with an 1100 HPLC system (Agilent Technologies, Santa Clara, CA, USA) equipped with an MS Detector 6110 single quadrupole. Selected ion monitoring of the molecular ion ([M] + 1) values for each compound and their deuterated homologs was used, as previously described [51].

### 2.6. Western Blot Assays for CB1R, CB2R, COX-2, PPARα, GFAP, and Iba1

Tissue homogenates were prepared from eight rats per group (SHAM-vehicle, BCCAO/R-vehicle, SHAM-KAM, BCCAO/R-KAM) in 2% sodium dodecyl sulfate (SDS) with protease inhibitors (cOmplete, Mini Protease Inhibitor Cocktail Tablets, Roche, Basel, Switzerland). Protein concentrations were measured using the Lowry protein assay [52]. For each sample, 40 μg of protein was separated by SDS-polyacrylamide gel electrophoresis (SDS-PAGE) (NuPAGE 4–12% Bis-Tris Gel Midi, Life Technologies, Waltham, MA USA) and transferred to a PVDF membrane (Amersham Hybond^TM^-P, GE Healthcare, Little Chalfont, UK). Membranes were blocked in TBS-T containing 5% milk and incubated overnight at 4 °C with the following rabbit polyclonal antisera: CB1R (Cat#258003, Synaptic System, Göttingen, Germany; 1:500), CB2R (Cat#101550 Cayman Chemical, Ann Arbor, Mi, USA; 1:1000), COX-2 (residues 570–598; Cat#160106, Cayman Chemical Ann Arbor, Mi, USA; 1:200), PPARα (Cat#PA1-822A, Thermo Scientific, Waltham, MA, USA; 1:1000), GFAP (Cat#Z0334, Dako, Glostrup, Denmark; 1:4000), and Iba1 (Cat#019-19741, Waco Pure Chemical Industries, Ltd., Osaka, Japan; 1:1000). After rinsing, blots were incubated with peroxidase-conjugated goat anti-rabbit serum (Sigma Aldrich; 1:10,000). Glyceraldehyde 3-phosphate dehydrogenase (GAPDH) (mouse monoclonal, Cat#MAB374, EMD Millipore, Darmstadt, Germany; 1:1000) was used as a loading control with a peroxidase-conjugated goat anti-mouse secondary antiserum (EMD Millipore, Darmstadt, Germany; 1:5000) as a secondary antiserum. To assess non-specific staining, blots were stripped and incubated with the secondary antiserum only. Protein bands were visualized using the ECL and an ImageQuant LAS 4000. Ratios of target proteins to GAPDH were calculated and O.D. values quantified with Li-Cor Image Studio Lite Software (LICORbio GmbH, Bad Homburg, Germany).

### 2.7. Immunohistochemistry for GFAP and Iba1

Coronal serial sections (14 μm thick) of the frontal and temporal-occipital cortex were cut with a cryostat, collected on chrome alum-gelatin-coated slides and processed by the avidin–biotin–peroxidase complex (ABC) technique. Brains of SHAM-vehicle (*n* = 8) and BCCAO/R-vehicle (*n* = 8) rats, as well as those of SHAM-KAM (*n* = 8) and BCCAO/R-KAM (*n* = 8) rats were processed in pairs on the same slide.

Slides were first treated with 0.1% phenylhydrazine in phosphate-buffered saline (PBS) containing 0.2% Triton X-100 (PBS/T) to inhibit endogenous peroxidase activity. They were then incubated with 20% normal goat serum (Cat# S-1000, Vector Labs Inc., Burlingame, CA, USA) for 1 h at room temperature. Rabbit polyclonal antibodies against COX-2 (residues 570–598; Cat# 160106, Cayman Chemical Ann Arbor, MI, USA), diluted 1:300, and against Iba1 (Cat# 019-19741, Wako Pure Chemical Industries, Ltd., Osaka, Japan), diluted 1:1000, served as the primary antibodies. A biotin-conjugated goat anti-rabbit serum (Cat# BA-1000, Vector Labs Inc., Burlingame, CA, USA), diluted 1:400, was used as the secondary antibody. The reaction product was visualized using the ABC (Cat# G011-61, Bio Spa Div. Milan, Italy), diluted 1:250, followed by development with a chromogen solution containing 0.05% 3,3′-diaminobenzidine (Sigma Aldrich, St. Louis, MO, USA), 0.04% nickel ammonium sulfate and 0.01% hydrogen peroxide in 0.1 M phosphate buffer, pH 7.3. All antisera and the ABC reagent were diluted in PBS/T. Incubation with primary antibodies was performed overnight at 4 °C. Incubations with the secondary antibody and ABC reagent lasted 60 min and 40 min, respectively, at room temperature. Negative controls were prepared by incubating tissue sections in parallel with PBS/T alone, by omitting the primary antibody, or by substituting the primary antibody with normal serum. Slides were examined using an Olympus BX61 microscope, and digital images were acquired with a Leica DFC450 C camera (Leica Microsystem, Wetzlar, Germany).

### 2.8. Statistical Analysis

**Figure 1:**
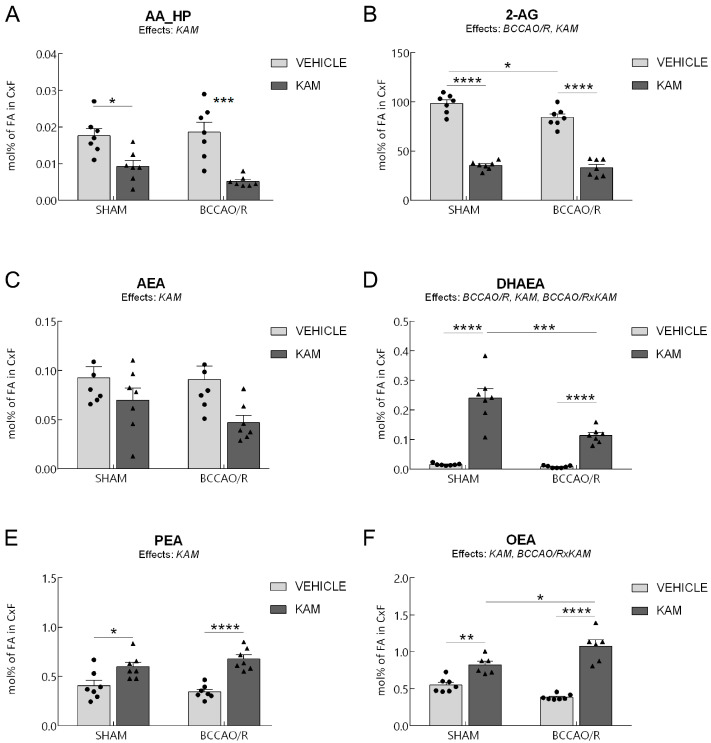
High-performance liquid chromatography (HPLC) and HPLC-mass spectrometry (HPLC-MS) were used to analyze the frontal cortex (CFX) of four experimental groups, SHAM-vehicle, SHAM-Kaempferol (KAM), bilateral common carotid artery occlusion followed by reperfusion (BCCAO/R)-vehicle, and BCCAO/R-KAM rats. (**A**) AA_HP, arachidonic acid hydroperoxides; (**B**) 2-AG, 2-arachidonoylglycerol; (**C**) arachidonoylethanolamide (AEA); (**D**) docosahexaenoylethanolamide (DHAEA); (**E**) palmitoylethanolamide (PEA); (**F**) oleoylethanolamide (OEA) concentrations, expressed as mol% of total fatty acid (FA). Results are presented as mean values (bars) and superimposed individual data points for vehicle- (*n* = 7 for both SHAM-vehicle and BCCAO/R-vehicle) (●) and KAM-treated (*n* = 7 for both SHAM-KAM and BCCAO/R-KAM) (▲) groups. Error bars represent the standard error of the mean (S.E.M.). Significance levels are indicated as follows: * *p* < 0.05; ** *p* < 0.005; *** *p* < 0.001; **** *p* < 0.0001. Refer to Table S1 for *F*- and *p*-values related to the effects of BCCAO/R and KAM pre-treatment, as well as their interaction.

**Figure 2:**
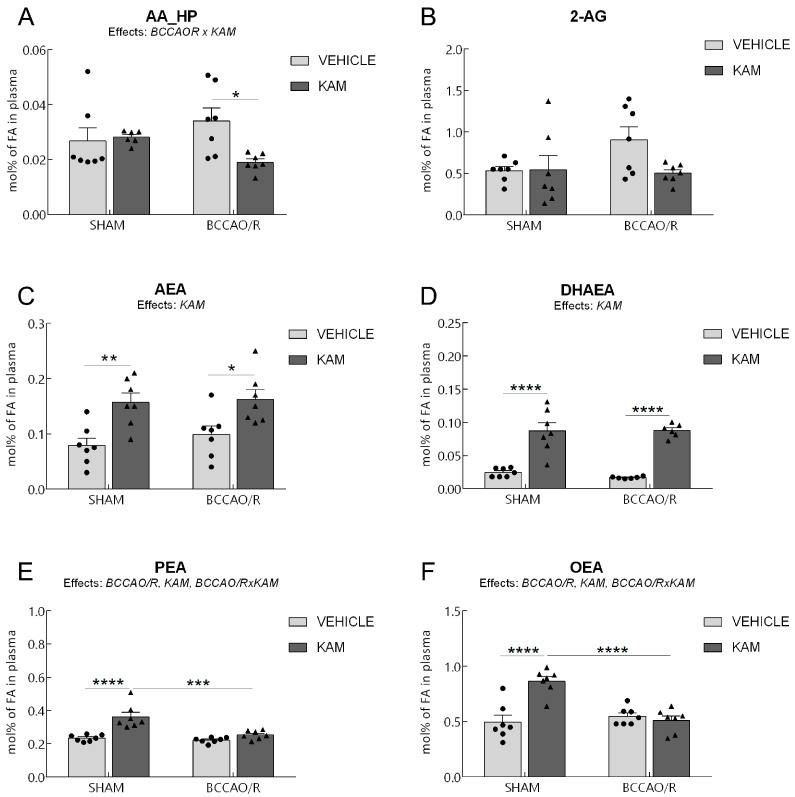
High-performance liquid chromatography (HPLC) and HPLC-mass spectrometry (HPLC-MS) were used to analyze the plasma of four experimental groups, SHAM-vehicle, SHAM-kaempferol (KAM), bilateral common carotid artery occlusion followed by reperfusion (BCCAO/R)-vehicle, and BCCAO/R-KAM rats. (**A**) AA_HP, arachidonic acid hydroperoxides; (**B**) 2-AG, 2-arachidonoylglycerol; (**C**) arachidonoylethanolamide (AEA); (**D**) docosahexaenoylethanolamide (DHAEA); (**E**) palmitoylethanolamide (PEA); (**F**) oleoylethanolamide (OEA) concentrations, expressed as mol% of total fatty acids (FA). Results are presented as mean values (bars) and superimposed individual data points for vehicle- (*n* = 7 for both SHAM-vehicle and BCCAO/R-vehicle)(●) and KAM-treated (*n* = 7 for both SHAM-KAM and BCCAO/R-KAM) (▲) rats. Error bars represent the standard error of the mean (S.E.M.). Significance levels are indicated as follows: * *p* < 0.05; ** *p* < 0.005; *** *p* < 0.001; **** *p* < 0.0001. Refer to Table S1 for *F*- and *p*-values related to the effects of BCCAO/R and KAM pre-treatment, as well as their interactions.

**Figure 3:**
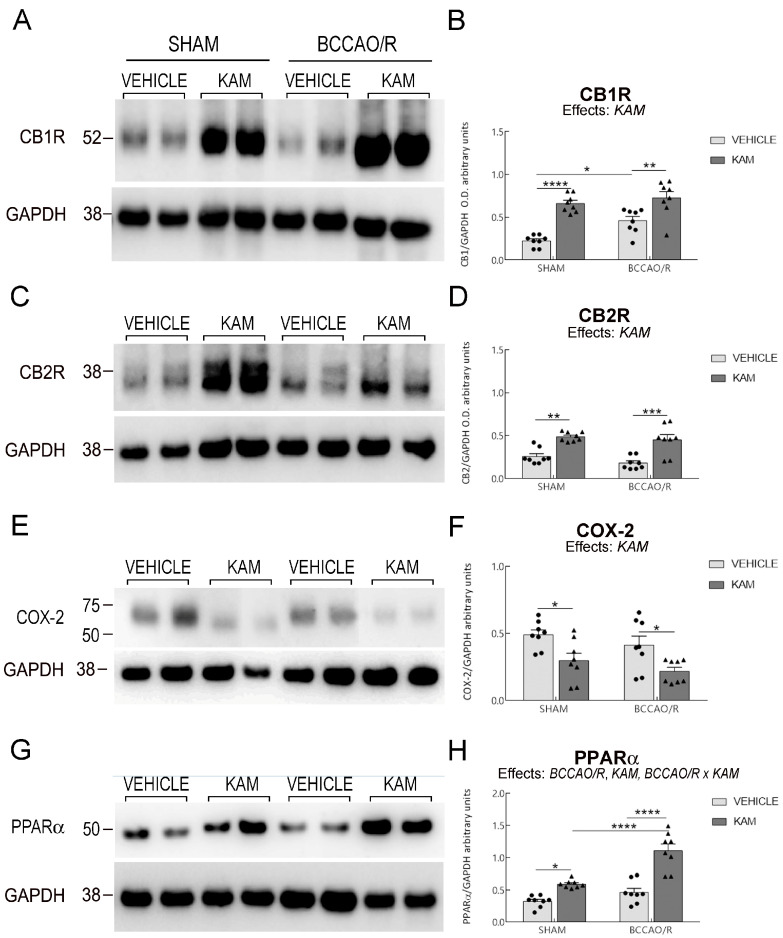
Western blot analysis was conducted to assess cannabinoid receptors CB1R (**A**,**B**) and CB2R (**C**,**D**), cyclooxygenase-2 (COX-2) (**E**,**F**), and peroxisome proliferator-activated receptor α (PPARα) (**G**,**H**) in the frontal cortex of four experimental groups: SHAM-vehicle, SHAM-kaempferol (KAM), bilateral common carotid artery occlusion followed by reperfusion (BCCAO/R)-vehicle, and BCCAO/R-KAM rats. (**B**,**D**,**F**,**H**) Densitometric analysis of the band gray levels, expressed as a percentage of the optical density (O.D.) ratio, was performed for immunostained bands of CB1R, CB2R, COX-2, and PPARα relative to GAPDH. Graphs represent mean values (bars) and superimposed individual data points for vehicle-treated (*n* = 8 for both SHAM-vehicle and BCCAO/R-vehicle) (●) and KAM-treated (*n* = 8 for both SHAM-KAM and BCCAO/R-KAM) (▲) rats. Error bars represent the standard error of the mean (S.E.M.). Significance levels are indicated as follows: * *p* < 0.05; ** *p* < 0.005; *** *p* < 0.001; **** *p* < 0.0001. Refer to Table S4 for *F*- and *p*-values related to the effects of BCCAO/R, KAM pre-treatment, and their interactions.

**Figure 4:**
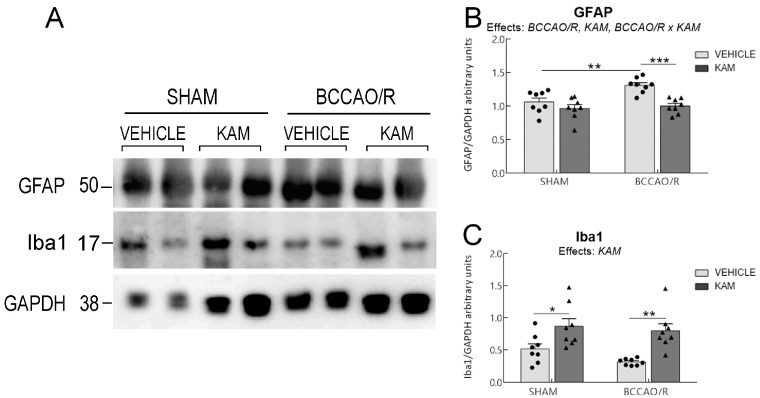
Western blot analysis (**A**) of glial fibrillary acidic protein (GFAP) and ionized calcium-binding adaptor molecule 1 (Iba1) in the frontal cortex of sham-operated and rats subjected to bilateral common carotid artery occlusion followed by reperfusion (BCCAO/R). Both vehicle-treated and kaempferol (KAM) pre-treated groups are shown. (**B**,**C**) Densitometric analysis, with band gray levels expressed as the percentage of the optical density (O.D.) ratio of GFAP- and Iba1-immunostained bands to GAPDH. Graphs represent mean values (bars) and superimposed individual data points for vehicle-treated (*n* = 8 for both SHAM-vehicle and BCCAO/R-vehicle) (●) and KAM-treated (*n* = 8 for both SHAM-KAM and BCCAO/R-KAM) (▲) rats. Error bars indicate the standard error of the mean (S.E.M.). Significance level is indicated as follows: * *p* < 0.05; ** *p* < 0.005; *** *p* < 0.001. Refer to Table S4 for *F*- and *p*-values related to the effects of BCCAO/R, KAM pre-treatment, and their interactions.

Data from the four experimental groups (SHAM-vehicle, BCCAO/R-vehicle, SHAM-KAM, and BCCAO/R-KAM) are shown in Figure 1, Figure 2, Figure 3 and Figure 4 and Figures S1 and S2 as mean ± standard error of the mean (S.E.M.). A two-way analysis of variance (ANOVA) was performed using GraphPad Prism 8.03 for Windows (GraphPad Software, La Jolla, CA, USA) to evaluate two main factors, BCCAO/R (sham operation versus BCCAO/R) and KAM treatment (vehicle versus KAM). When two-way ANOVA revealed statistically significant effects, multiple pairwise comparisons were conducted using Tukey’s post hoc test and the obtained multiplicity-adjusted *p*-values for each comparison are denoted by asterisks in the graphs of Figure 1, Figure 2, Figure 3 and Figure 4.

## 3. Results

Analysis of lipid extracts from the cerebral tissue (Tables S1 and S2; Figure 1) and plasma (Tables S1 and S3; Figure 2), as well as WB analysis of the tissue homogenates for protein markers (Table S4; Figure 3 and Figure 4) revealed that molecular changes following KAM pre-treatment could be observed only in the frontal cortex, to which Figure 1, Figure 3 and Figure 4 refer. No statistically significant changes were detected in the temporal-occipital cortex (Figures S1 and S2).

### 3.1. Analysis of Fatty Acid Profiles, Endocannabinoids, and Congeners in Frontal Cortex

The two-way ANOVAs revealed an effect of the BCCAO/R on tissue concentrations of 2-AG and DHAEA. Additionally, KAM administration influenced levels of AA oxidative metabolites (AA_HP), eCBs, and NAEs including DHAEA, PEA, and OEA in the frontal cortex of both sham-operated and BCCAO/R rats, as summarized in Table S1 and shown in Figure 1. The analysis of fatty acid profiles in brain tissues revealed no statistically significant differences (Table S2).

Regarding the AA_HP (Figure 1A), pairwise comparisons using Tukey’s test between animals in basal conditions showed that the sham-KAM rats had lower AA_HP levels than sham-vehicle rats (−46.7%; post hoc adjusted *p* = 0.021). Further post hoc tests also showed that, after BCCAO/R, AA_HP levels were lower in BCCAO/R-KAM vs. BCCAO/R-vehicle rats (−72.3%; post hoc adjusted *p* = 0.0002).

Regarding the eCBs and their congeners, pairwise contrasts between animals under basal conditions showed that 2-AG concentrations were lower in SHAM-KAM vs. SHAM-vehicle rats (−64%; post hoc adjusted *p* < 0.0001). In contrast, DHAEA (+1473%; post hoc adjusted *p* < 0.0001), PEA (+48%; post hoc adjusted *p* < 0.0001), and OEA (+50%; post hoc adjusted *p* = 0.0041) were higher in the SHAM-KAM vs. the SHAM-vehicle rats (Figure 1D–F). Pairwise comparisons with Tukey’s test also showed that the BCCAO/R per se caused a decrease in 2-AG concentrations (−15%; post hoc adjusted *p* = 0.0184) in BCCAO/R-vehicle vs. SHAM-vehicle animals (Figure 1B). Additional post hoc tests also showed differences in both groups of BCCAO/R rats; thus, the concentrations of AA derivative 2-AG (−61%; post hoc adjusted *p* < 0.0001) were lower in BCCAO/R-KAM vs. BCCCAO/R-vehicle rats, whereas DHAEA (+1384%; post hoc adjusted *p* = 0.0008), PEA (+98%; post hoc adjusted *p* = 0.0008), and OEA (+180%; post hoc adjusted *p* < 0.0001) were higher in BCCAO/R-KAM vs. BCCAO/R-vehicle rats. Finally, Tukey’s test also showed the impact of the BCCAO/R per se in KAM-pre-treated animals (Table S1; Figure 1); in fact, significantly lower DHAEA levels (−53%; post hoc adjusted *p* < 0.0001) were found in BCCAO/R-KAM vs. SHAM-KAM rats; by contrast, higher OEA (+31%; post hoc adjusted *p* = 0.0115) were found in BCCAO/R-KAM vs. SHAM-KAM rats. Consistently, two-way ANOVAs also revealed a statistically significant BCCAO/R *x* KAM interaction for DHAEA and OEA (Table S1; Figure 1D,F).

### 3.2. Analysis of Fatty Acid Profiles, Endocannabinoids, and Congeners in Plasma

The two-way ANOVAs revealed an effect of the BCCAO/R on the plasmatic concentrations of PEA and OEA, as well as an effect of KAM administration on AEA and eCB congeners DHAEA, PEA, and OEA, as summarized in Table S1 and shown in Figure 2. The analysis of fatty acid profiles in plasma revealed no statistically significant differences (Table S3). Pairwise comparisons of animals in basal conditions showed that the SHAM-KAM rats had higher concentration levels of AEA (+100%; post hoc adjusted *p* = 0.0093), DHAEA (+257%; post hoc adjusted *p* < 0.0001), PEA (+56%; post hoc adjusted *p* < 0.0001), and OEA (+ 75%; post hoc adjusted *p* < 0.0001) than the SHAM-vehicle rats (Figure 2C,E,F). Additional post hoc analyses indicated differences between the two BCCAO/R groups; thus, after KAM pre-treatment, lower levels of AA_HP could be found in BCCAO/R-KAM vs. BCCAO/R-vehicle rats (−44%; post hoc adjusted *p* = 0.0261) (Figure 2A). In contrast, higher concentrations of AEA (+64%; post hoc adjusted *p* = 0.0426) and DHAEA (+426%; post hoc adjusted *p* < 0.0001) were found in BCCAO/R-KAM than in BCCAO/R-vehicle animals. Tukey’s test also showed a significant effect of the BCCAO/R per se in KAM-pre-treated animals (Table S1; Figure 2); in fact, significant decreases in PEA (−30%; post hoc adjusted *p* < 0.0001) and OEA (−41%; post hoc adjusted *p* < 0.0001) were found in BCCAO/R-KAM vs. SHAM-KAM rats (Figure 2E,F). Moreover, the two-way ANOVAs revealed statistically significant BCCAO/R *x* KAM interactions for AA_HP, PEA, and OEA (Table S1; Figure 2).

### 3.3. Analysis of CB1R, CB2R, COX-2, PPARα, GFAP, and Iba1 in Frontal Cortex

The effects of KAM administration on the relative protein levels of CB1R and CB2R, COX-2, PPARα, GFAP, and Iba1 in the frontal cortex are reported in Table S4 and graphically illustrated in Figure 3 and Figure 4. The two-way ANOVAs revealed statistically significant effects of KAM for all examined protein markers and of BCCAO/R for CB1R, PPARα, and GFAP, as well as BCCAO/R *x* KAM interaction for PPARα and GFAP (Table S4; Figure 3 and Figure 4). The effect of KAM pre-treatment was evident in the pair-wise comparison; thus, the sham-KAM vs. sham-vehicle rats showed higher relative levels of CB1R (+195%; post hoc adjusted *p* < 0.0001), CB2R (+89%; post hoc adjusted *p* = 0.0012), PPARα (+83%; post hoc adjusted *p* = 0.0263), Iba1 (+41%; post hoc adjusted *p* = 0.0446), and lower relative levels of COX-2 (−39%; post hoc adjusted *p* = 0.048) (Table S4; Figure 3). Additional pairwise contrasts between the BCCAO/R rat groups showed higher relative levels of CB1R (+58%; post hoc adjusted *p* = 0.0035), CB2R (+150%; post hoc adjusted *p* = 0.0002), PPARα (+139%; post hoc adjusted *p* < 0.0001), and Iba1 (+61%; post hoc adjusted *p* < 0.0033) in BCCAO/R-KAM vs. the BCCAO/R-vehicle animals, while revealing a decrease in the relative levels of COX-2 (−10%; post hoc adjusted *p* = 0.0434) and GFAP (−31%; post hoc adjusted *p* = 0.0007) (Table S4; Figure 3 and Figure 4). The post hoc analysis also revealed an effect of the BCCAO/R per se; thus, CB1R relative levels (+105%; post hoc adjusted *p =* 0.0112) increased in BCCAO/R-vehicle rats compared to SHAM-vehicle rats, and PPARα relative levels (+88%; post hoc adjusted *p* < 0.0001) in the BCCAO/R-KAM vs. the SHAM-KAM rats (Table S4; Figure 3). Consistently, a statistically significant BCCAO/R *x* KAM interaction was observed for PPARα (*p* = 0.0056) and GFAP (*p* = 0.0391) (Table S4).

### 3.4. Immunohistochemistry for COX-2 and Iba1

**Figure 5:**
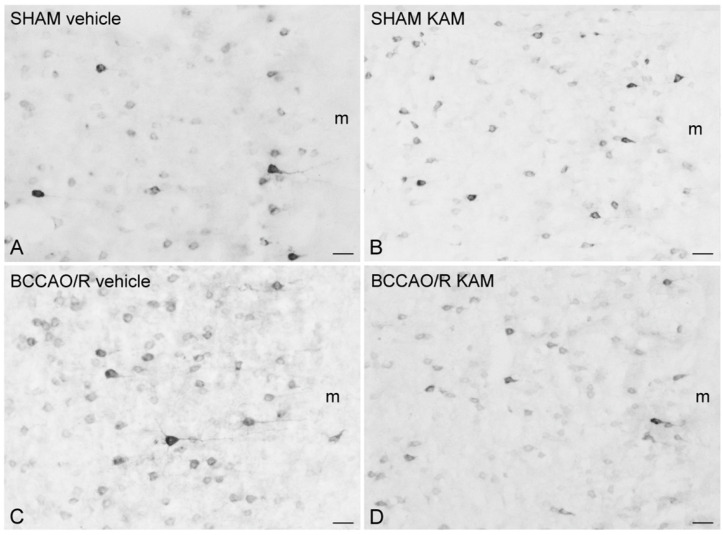
Cyclooxygenase-2 (COX-2)-like immunoreactivity is shown in representative coronal sections of the frontal cortex (prelimbic/infralimbic cortex) from SHAM-operated and bilateral common carotid artery occlusion followed by reperfusion (BCCAO/R) rats. Animals were pre-treated with either vehicle (**A**,**C**) or kaempferol (KAM) (**B**,**D**). Positive cell bodies are primarily localized in the superficial layers of the cortex. Each panel represents observations from four rats per group. m, molecular layer. Scale bars: (**A**–**D**) = 25 μm.

**Figure 6:**
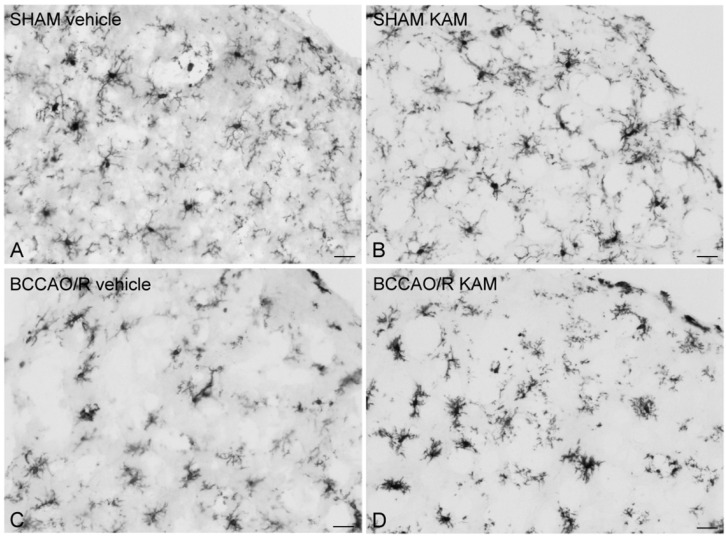
Ionized calcium-binding adaptor molecule 1 (Iba1)-like immunoreactive microglial cells in representative coronal sections of the frontal cortex (anterior cingulate cortex) of sham-operated and rats subjected to bilateral common carotid artery occlusion followed by reperfusion (BCCAO/R). Animals were pre-treated with either vehicle alone (**A**,**C**) or kaempferol (KAM) (**B**,**D**). Each panel represents data from four rats per group. Scale bars: (**A**–**D**) = 25 μm.

To compare the molecular changes observed through Western blot analysis with tissue morphology, we performed COX-2 and Iba1 immunostainings on rat brain sections using the same antibodies applied in the Western blot experiments. The immunoreactivities for the examined markers were localized to neuronal structures throughout the rostro-caudal extent of the brain (Figure 5 and Figure 6).

In the frontal cortex, COX-2-like immunoreactivity appeared as neuronal perikarya and processes exhibiting varying levels of intracytoplasmic immunostaining (Figure 5). COX-2-immunolabeled neurons were commonly observed in the cortical layers II/III and V. Generally, the brains of vehicle-treated rats showed higher staining intensity and density of labeled structures in the BCCAO/R group compared to the SHAM-operated rats (Figure 5A,C). However, this difference was not apparent in the KAM-pre-treated rats (Figure 5B,D).

Iba1-labeled microglial cells were observed throughout the cortical layers (Figure 6). The spacing between the Iba1-positive cell bodies was relatively constant in the SHAM-operated rats (Figure 6A,B), while it appeared somewhat deranged in the cortex of BCCAO/R rats (Figure 6C,D). Interestingly, the processes arising from the Iba1-labeled microglial cells appeared to be distributed in a relatively uniform fashion in the cortex of SHAM-vehicle rats (Figure 6A), such that each microglial cell with its processes had its own domain that rarely overlapped with adjacent microglial cells.

## 4. Discussion

This study demonstrated that a single acute administration of KAM confers robust neuroprotection against the molecular alterations induced by the BCCAO/R, as evidenced by significant prevention of both cerebral and plasmatic changes. Importantly, this neuroprotective effect is observed even after a single oral dose administered prior to the BCCAO/R-induced insult, highlighting the rapid efficacy of KAM. The observed benefits are most pronounced in the frontal cortex, a region highly susceptible to hypoperfusion/reperfusion due to its vascularization by internal carotid artery branches. KAM’s effects encompass both anti-inflammatory and anti-oxidative actions, with broad modulation of tissue lipid composition and the activity of lipid-sensing receptors. These findings provide new insight into the acute, region-specific action of KAM and suggest a previously underappreciated potential for rapid intervention in ischemic events.

### 4.1. Key Effects of KAM Administration

Thus, KAM (a) decreased the levels of the oxidative products of AA in the brain tissue both under basal conditions and following the BCCAO/R; (b) modulated the ECS in both SHAM conditions and following the BCCAO/R by decreasing levels of 2-AG and increasing those of DHAEA, PEA, and OEA in the brain tissue; (c) induced increases in CB1R, CB2R, and PPARα while decreasing COX-2 activation under both SHAM conditions and following the BCCAO/R; (d) modulated the glial cell response by decreasing the relative levels of GFAP and increasing those of Iba1; and (e) increased plasmatic levels of AEA, DHAEA, PEA, and OEA.

### 4.2. KAM in Preclinical and Clinical Research

The current data corroborate previous reports on the progression of cerebral damage after transient cerebral hypoperfusion and reperfusion, substantiating the provided evidence that metabolic disturbances induced by BCCAO/R occur prior to the onset of oxidative stress and neuroinflammation [7,16,53,54]. However, the present study advances existing knowledge by demonstrating that targeted intervention with KAM can disrupt this sequence, preventing downstream inflammatory and oxidative cascades. This underlying insight distinguishes our findings from earlier work and positions KAM as a promising modulator of early pathological events in cerebral hypoperfusion/reperfusion.

Recent studies reported the beneficial effects of KAM in preclinical models of age-related diseases [23,29,55,56]. Additionally, epidemiological and clinical research supports the efficacy of KAM-containing foods for a range of pathological conditions, including cardiovascular, neurodegenerative, and metabolic diseases [24,57,58]. Despite these advances, a critical gap persists regarding the discrete neuroprotective mechanisms of purified KAM, distinct from the complex bioactive matrix present in whole foods. Our findings address this gap by isolating the effects of acute KAM administration, providing direct evidence for its standalone therapeutic potential in the context of cerebral hypoperfusion/reperfusion.

#### Bioavailability of KAM and Translational Relevance in Human Studies

KAM is poorly absorbed when taken orally and has very low oral bioavailability; however, it is highly effective in vivo because it rapidly transforms into active metabolites [55,57,59]. In studies on rats, plasma concentrations of KAM following intravenous administration at doses of 10 mg/kg and 25 mg/kg decreased by 50% within 3–4 h and further declined after 12 h [60]. For oral doses of 100 mg/kg and 250 mg/kg, a similar 50% decrease in plasma concentration was observed within 1–2 h, as evidenced by portal blood collection [60], while only about 4% of the total amount of KAM ingested is excreted in urine [61].

From a translational point of view, recent data from humans demonstrate that glucuronide metabolites predominate after both intravenous and oral administrations, with absorption rates similar to those observed in the rat [62].

### 4.3. Molecular Markers and Mechanisms

Our Western blot results highlight that the preventive administration of KAM increases the relative protein levels of CBR and PPARα, while decreasing those of COX-2 and lipoperoxide concentrations, further suggesting that KAM may simultaneously activate multiple factors to counter the inflammatory response induced by BCCAO/R. Notably, PPARα, as part of its transcriptional function, stimulates the expression of genes related to mitochondrial and peroxisomal fatty acid β-oxidation [32,39,63].

#### 4.3.1. COX-2

In this study, we observed that COX-2 protein expression in the frontal cortex remained unchanged following the BCCAO/R, but pre-treatment with KAM significantly decreased COX-2 levels both under basal conditions and after BCCAO/R. Previous studies reported that COX-2 is upregulated in the BCCAO/R model [7] and in cerebral I/R models, and its inhibition has been shown to be neuroprotective [64,65,66].

Given our earlier findings of elevated lipid peroxidation in BCCAO/R rats [7], which indicates oxidative stress, we quantified oxidative metabolites derived from AA. Surprisingly, BCCAO/R did not increase levels of AA_HP and other enzymatic AA-derived eicosanoids. This suggests that COX-2 downstream cascade was not robustly activated at the eicosanoid synthesis level, even with COX-2 present in the tissue. In contrast, KAM significantly reduced the oxidized AA products in BCCAO/R animals, indicating an anti-inflammatory mechanism through the modulation of eicosanoid biosynthesis downstream of COX-2 [67].

#### 4.3.2. PPARα

In addition to lowering oxygenated AA metabolites, AA_HP, KAM also decreased levels of the endocannabinoid 2-AG, a bioactive lipid known to modulate synaptic and immune signaling. This suggests that KAM may broadly inhibit the release of AA or its incorporation into signaling lipids. However, levels of other NAEs, particularly the agonists PEA and OEA, significantly increased. Since KAM has been reported to act as a FAAH inhibitor, based on in vitro and in silico studies [68,69], we suggest that this likely indicates a shift towards the biosynthesis of the monounsaturated/saturated NAE derivatives, OEA and PEA, rather than a generalized suppression of NAE synthesis.

PEA and OEA are recognized as anti-inflammatory lipids that act via PPARα activation, which regulates genes implicated in inflammation resolution and oxidative stress. Furthermore PPARα activation increases PEA and OEA levels, which may further sustain PPARα activity [70,71]. Their increase implies that KAM initiates a PPARα–mediated neuroprotective transcriptional program. Moreover, the increase in DHAEA (or synaptamide) is noteworthy since DHAEA may support synaptic plasticity and exerts neuroprotective, anti-inflammatory effects [72,73].

Overall, these findings support a dual anti-inflammatory mechanism for KAM, i.e., suppression of COX-2-mediated eicosanoid production and reprogramming of the NAE pool towards PPARα agonists (PEA, OEA). This shift amplifies anti-inflammatory gene expression and helps preserve neuronal homeostasis [72].

### 4.4. Effects of KAM on Glial Markers

Our findings show that KAM pre-treatment significantly changed the glial marker expression in the brain, both under baseline conditions and after BCCAO/R. Specifically, KAM prevented the BCCAO/R-induced increase in GFAP. This suggests that KAM may regulate the vascular bed by modulating blood–brain barrier (BBB) permeability during inflammation or by adjusting cerebral vessel tone [74]. Although our study does not detail how glial cells contribute to KAM’s anti-inflammatory effects, further study of GFAP expression in tissue after a single KAM dose is warranted. According to the findings that KAM raised the levels of the anti-inflammatory NAEs, such as PEA, and lowered the oxidized metabolites from AA, it is furthermore interesting that in co-culture studies, Viader et al. [75] recently showed that neurons produce extracellular 2-AG, and astrocytes convert this to AA and return it to neurons. Therefore, identifying protein carriers or transporters in neuron-astrocyte lipid shuttling deserves future research. In contrast to the reduction in GFAP levels, KAM increased the microglial marker Iba1 in both sham and BCCAO/R groups. This aligns with reports that microglia activate within 10 min of BCCAO and reach peak response in 20 min, even without reperfusion [76]. Notably, the level of microglial response has been shown not to increase with longer survival after BCCAO, suggesting a possible neuroprotective effect, and that reperfusion can easily reverse it [76]. Our Iba1 immunostaining also suggests that KAM prevents microglial disruption seen after BCCAO/R.

### 4.5. Systemic Effects Observed in Plasma

In plasma, KAM pre-treatment in BCCAO/R rats significantly increased levels of DHAEA, mirroring changes observed in the cortex and indicating a systemic enhancement of neuroprotective lipid mediators.

Additionally, increases in plasma OEA and PEA were observed in SHAM-KAM but not in BCCAO/R-KAM rats. This discrepancy with levels observed in the cortex suggests that, under SHAM conditions, KAM mobilizes pools of peripheral PPARα ligands, while following injury, these substances may be redirected or taken up by the brain. This is consistent with findings that peripheral NAEs can cross the BBB [77,78] and modulate central inflammation as previously demonstrated [45]. Other mechanisms, such as KAM-induced PPARα-mediated upregulation of CD36 in the BBB [79], also warrant further investigation.

## 5. Conclusions

In summary, our study reveals that KAM mediates a multifaceted neuroprotective response in a well-established model of cerebral hypoperfusion and reperfusion. Although BCCAO/R alone did not significantly elevate COX-2 protein or its oxidative lipid products, KAM administration led to a pronounced reduction in both COX-2 protein levels and the generation of downstream eicosanoids. The most striking finding is KAM’s ability to recalibrate both the cerebral and systemic lipidome—marked by reductions in pro-inflammatory endocannabinoids (2-AG, AEA) and elevations in PPARα agonists (PEA, OEA) and DHAEA. This comprehensive lipidomic shift underlies KAM’s dual anti-inflammatory and neuroprotective actions, operating through both enzymatic pathways and PPARα-driven gene transcription. By directly intervening in the early pathophysiological sequence of cerebral hypoperfusion/reperfusion, KAM emerges as a novel therapeutic candidate for targeting neuroinflammation and maintaining neurovascular homeostasis. These results contextualize KAM’s effects within the broader literature and underscore the translational relevance and novelty of our work.

## Data Availability

The data presented in the current study are available from the corresponding author on reasonable request.

## Supplementary Materials

The following supporting information and original images of western blot can be downloaded at https://www.mdpi.com/article/10.3390/biom15101440/s1, Figure S1: Western blot analysis of cannabinoid receptors CB1R (A,B) and CB2R (C,D), cyclooxygenase-2 (COX-2) (E,F), and peroxisome-proliferator activated receptor α (PPARα) (G,H) in the temporal-occipital cortex of four experimental groups: SHAM-vehicle, SHAM- kaempferol (KAM), bilateral common carotid artery occlusion followed by reperfusion (BCCAO/R)-vehicle and BCCAO/R-KAM rats. (B,D,F,H) Densitometric analysis of the band gray levels, expressed as a percentage of the optical density (O.D.) ratio, was performed for immunostained bands of CB1R, CB2R, COX-2, and PPARα relative to GAPDH. Graphs represent mean values (bars) and superimposed individual data points for vehicle-treated (*n* = 8 for both SHAM-vehicle and BCCAO/R-vehicle) (●) and KAM-treated (*n* = 8 for both SHAM-KAM and BCCAO/R-KAM) (▲) rats. Error bars represent the standard error of the mean (S.E.M.). Statistical differences between groups were evaluated using two-way ANOVA. Refer to Table S1 for F- and p-values related to the effects of BCCAO/R, KAM pre-treatment, and their interaction. Figure S2: Western blot analysis (A) of glial fibrillary acidic protein (GFAP) and ionized calcium-binding adaptor molecule 1 (Iba1) in the temporal-occipital cortex of sham-operated and rats subjected to bilateral common carotid artery occlusion followed by reperfusion (BCCAO/R). Both vehicle-treated and kaempferol (KAM) pre-treated groups are shown. (B,C) Densitometric analysis, with band gray levels expressed as the percentage of the optical density (O.D.) ratio of GFAP- and Iba1-immunostained bands to GAPDH. Graphs represent mean values (bars) and superimposed individual data points for vehicle-treated (*n* = 8 for both SHAM-vehicle and BCCAO/R-vehicle) (●) and KAM-treated (*n* = 8 for both SHAM-KAM and BCCAO/R-KAM) (▲) rats. Error bars indicate the standard error of the mean (S.E.M.). Statistical differences between groups were evaluated using two-way ANOVA. Refer to Table S1 for *F*- and *p*-values related to the effects of BCCAO/R, KAM pre-treatment, and their interaction. Table S1: Statistical data, F-Value and p-Value, performed on endocannabinoids (eCBs) and N-acylethanolamines (NAEs) concentrations (expressed as mol% of total fatty acid) in the frontal cortex and plasma of the four experimental groups: SHAM-vehicle, SHAM-KAM, bilateral common carotid artery occlusion followed by reperfusion (BCCAO/R)-vehicle and BCCAO/R-kaempferol (KAM) rats. F-Values and significance levels from two-way ANOVAs performed on data obtained by means of HPLC and HPLC-MS. Table S2: Main fatty acid (FA) concentrations in the frontal cortex from the four experimental groups: SHAM-vehicle, SHAM-KAM, bilateral common carotid artery occlusion followed by reperfusion (BCCAO/R)-vehicle and BCCAO/R-kaempferol (KAM) rats. Quantitative data, expressed as mol% of total FA and presented as mean ± SEM (*n* = 7 per group), were obtained using HPLC and GC. Table S3: Main fatty acid (FA) concentrations in plasma from the four experimental groups: SHAM-vehicle, SHAM-KAM, bilateral common carotid artery occlusion followed by reperfusion (BCCAO/R)-vehicle, and BCCAO/R-kaempferol (KAM) rats. Quantitative data, expressed as mol% of total FA and presented as mean ± SEM (*n* = 7 per group), were obtained using HPLC and GC. Table S4: Statistical data, F-Value and p-Value, performed on relative protein levels of CB1R, CB2R, COX-2, PPARα, and glial markers GFAP and Iba1 obtained in homogenates of frontal cortex and temporal-occipital cortex from the four experimental groups: SHAM-vehicle, SHAM-KAM, bilateral common carotid artery occlusion followed by reperfusion (BCCAO/R)-vehicle and BCCAO/R-kaempferol (KAM) rats. *F*-Values and significance levels from two-way ANOVAs performed on data obtained using Western Blot.

## Abbreviations

The following abbreviations are used in this manuscript:

AA	arachidonic acid
AA_HP	arachidonic acid_hydroperoxides
AEA	arachidonoylethanolamide
2-AG	2-arachidonoylglycerol
BCCAO	Bilateral Common Carotid Artery Occlusion
CBR	cannabinoid receptor
CB1R	cannabinoid receptor type 1
CB2R	cannabinoid receptor type 2
COX-2	cyclooxygenase-2
DHA	docosahexaenoic acid
DHAEA	docosahexaenoylethanolamide
eCBs	endocannabinoids
ECS	endocannabinoid system
FA	fatty acids
FAAH	fatty acid amide hydrolase
GFAP	glial fibrillary acidic protein
Iba1	ionized calcium-binding adapter molecule 1
KAM	kaempferol
NAEs	*N*-acylethanolamines
OEA	oleoylethanolamide
PEA	palmitoylethanolamide
PUFA	polyunsaturated fatty acids
PPARα	peroxisome proliferator-activated receptor alpha
PVDF	polyvinylidene fluoride
SFA	saturated fatty acids
TBS-T	(Tris base, sodium chloride, Tween 20)
UFA	unsaturated fatty acids
