# Supplementary material for: Kaempferol Regulates Lipid Homeostasis, Endocannabinoid System, and PPARα in Rat Cerebral Cortex Following BCCAO/R
Source: Biomolecules. 2025 Oct 11;15(10):1440. doi: 10.3390/biom15101440 (PMC12563104; doi:10.3390/biom15101440)
Supplement: Supplementary file 1 [file biomolecules-15-01440-s001.zip › biomolecules-3839299-Original WB images.pdf]

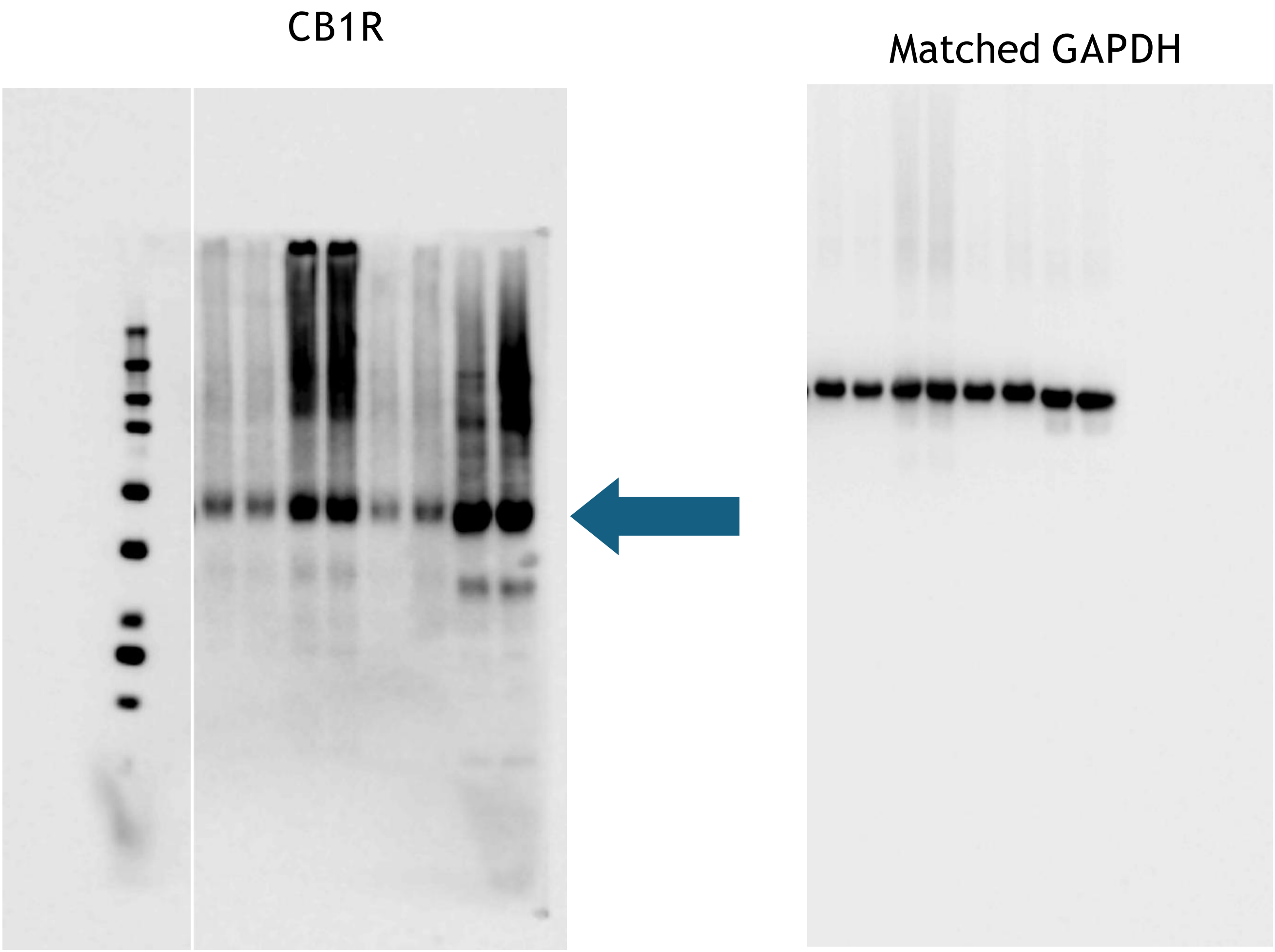

Original uncropped images of western blot analysis shown in Figure 3**:** cannabinoid receptors CB1R (and matched GAPDH) in the frontal cortex

CB2R

Matched GAPDH

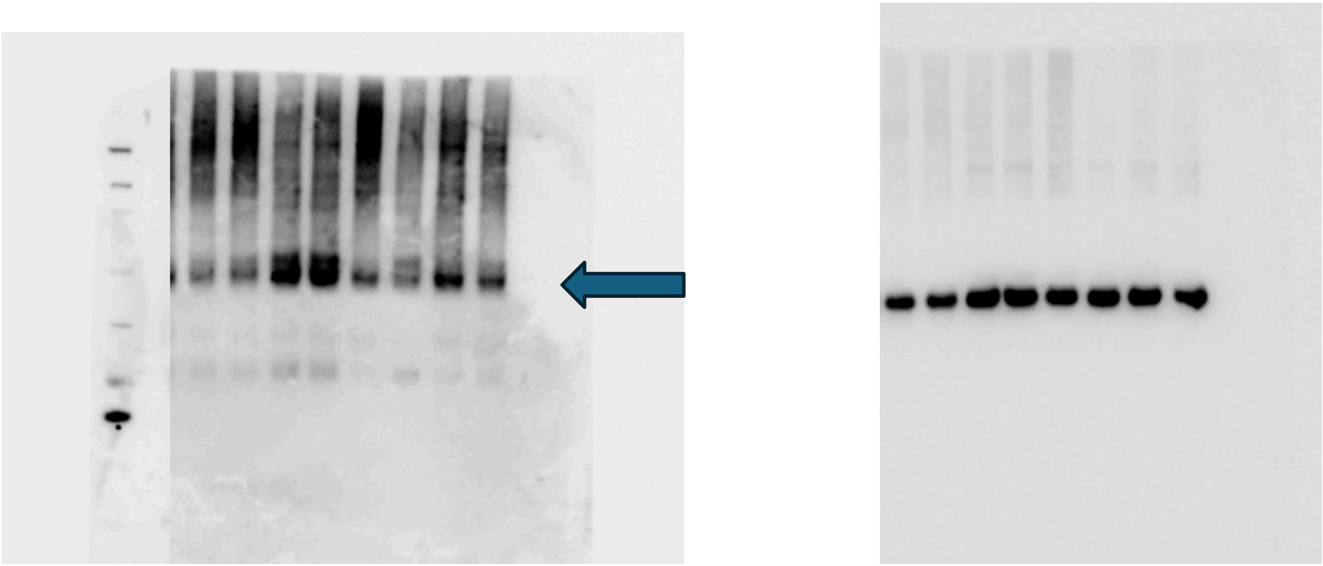

Original uncropped images of western blot analysis shown in Figure 3**:** cannabinoid receptors CB2R (and matched GAPDH) in the frontal cortex

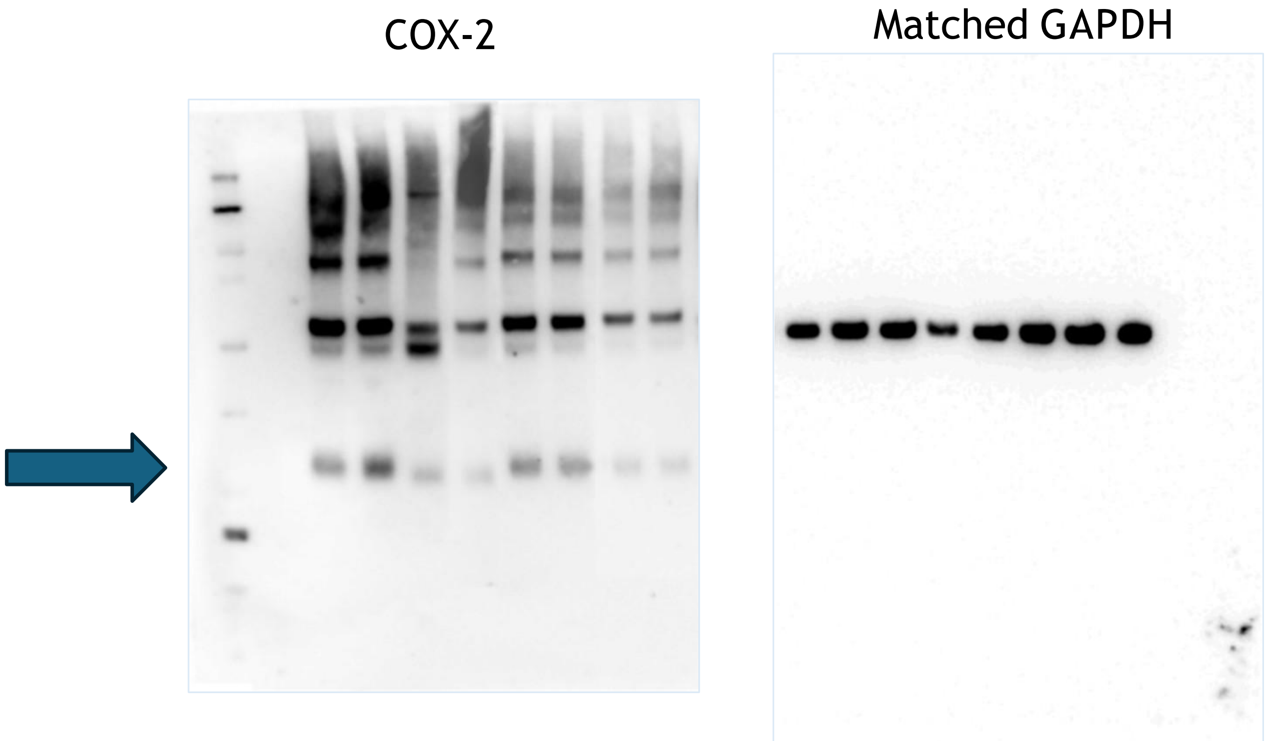

Original uncropped images of western blot analysis shown in Figure 3**:** COX-2 (and matched GAPDH) in the frontal cortex

PPARα

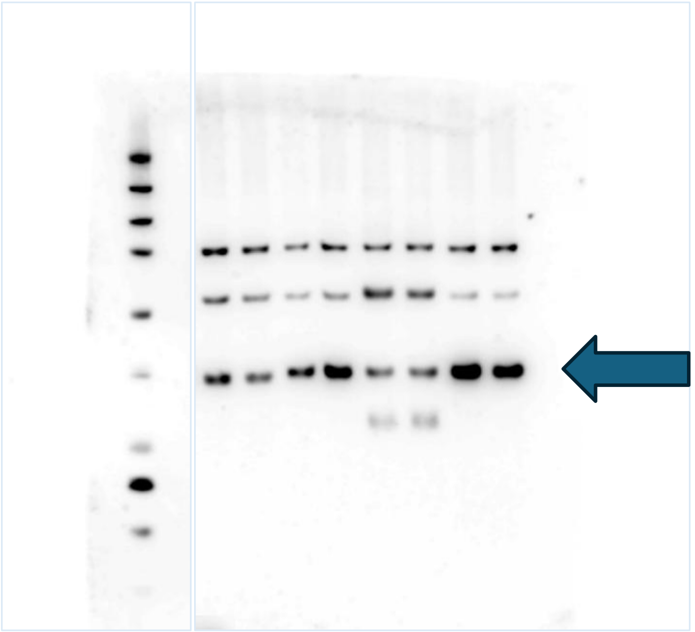

Matched GAPDH

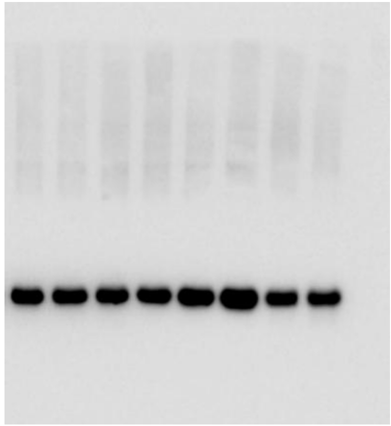

Original uncropped images of western blot analysis shown in Figure 3**:** PPARα (and matched GAPDH) in the frontal cortex

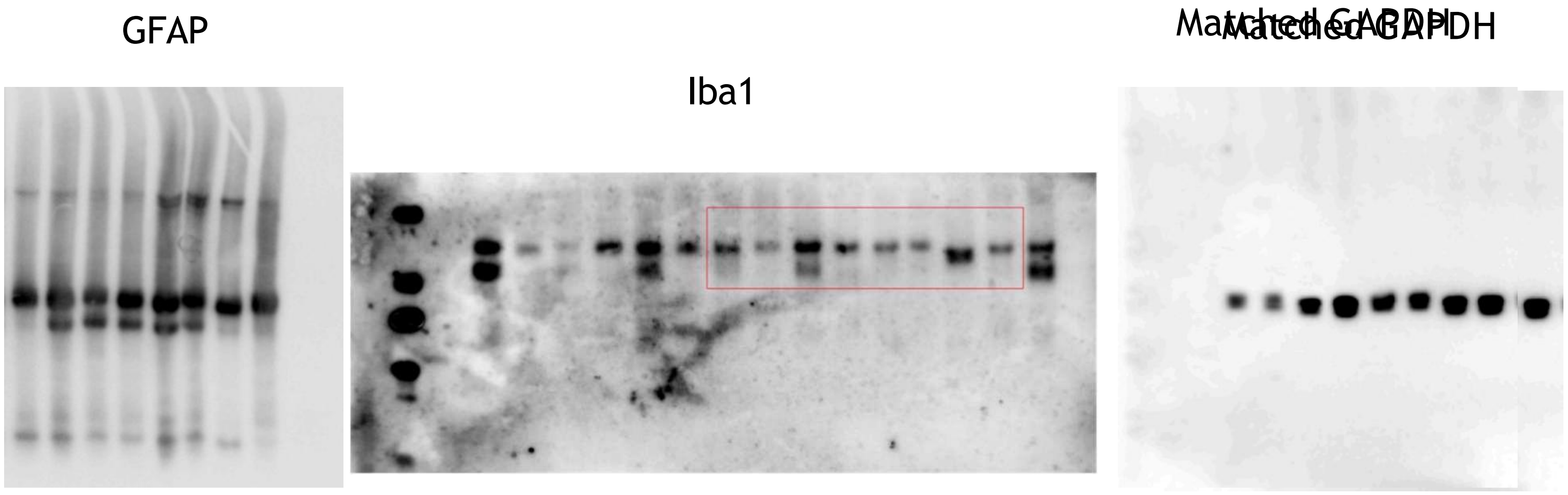

Original uncropped images of western blot analysis shown in Figure 4**:** glial markers GFAP and Iba1 (and matched GAPDH) in the frontal cortex

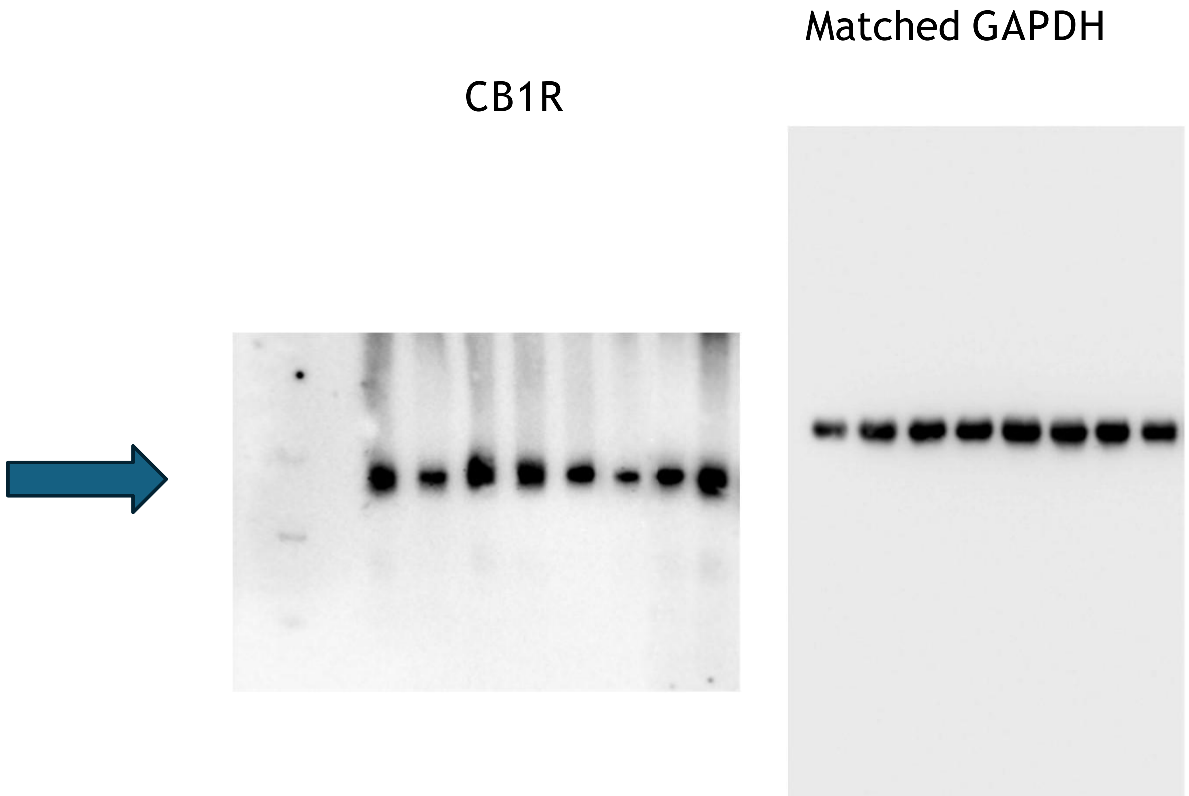

Original uncropped images of western blot analysis shown in Figure S3**:** cannabinoid receptors CB1R (and matched GAPDH) in the temporal-occipital cortex

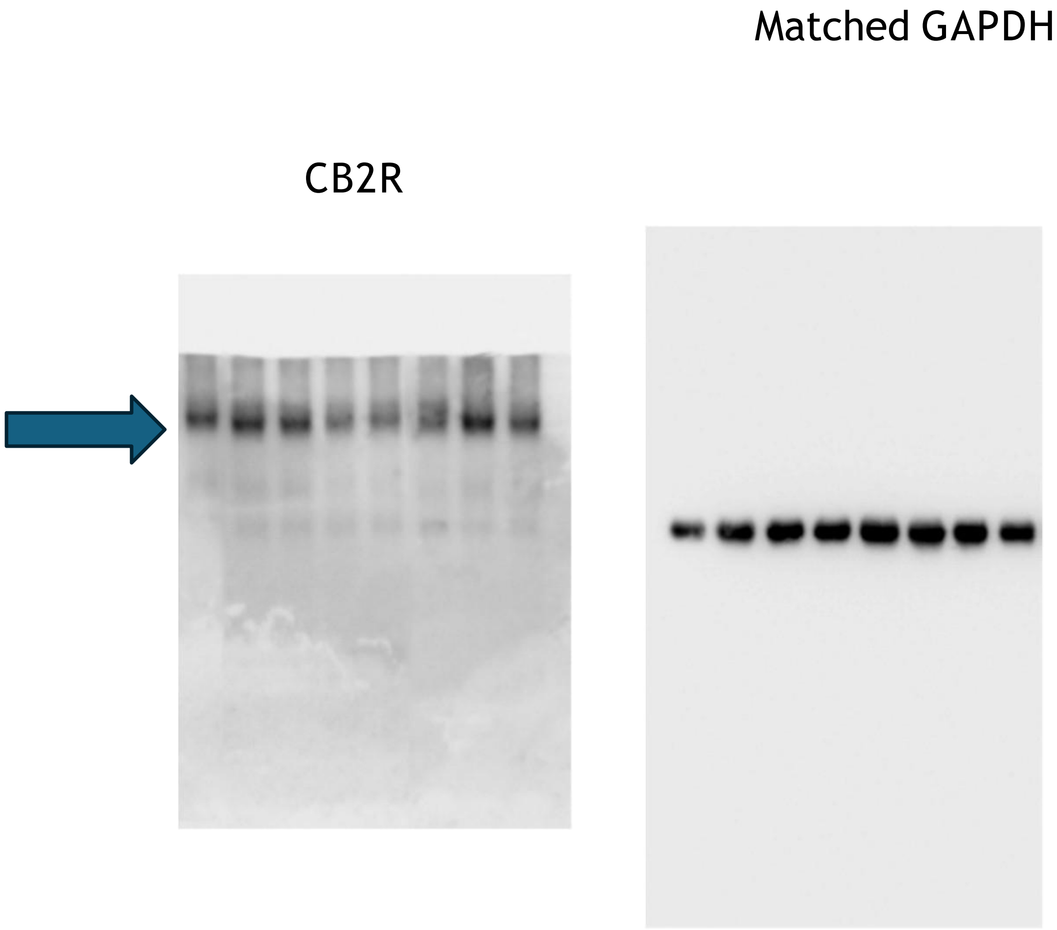

Original uncropped images of western blot analysis shown in Figure S3**:** cannabinoid receptors CB2R (and matched GAPDH) in the temporal-occipital cortex

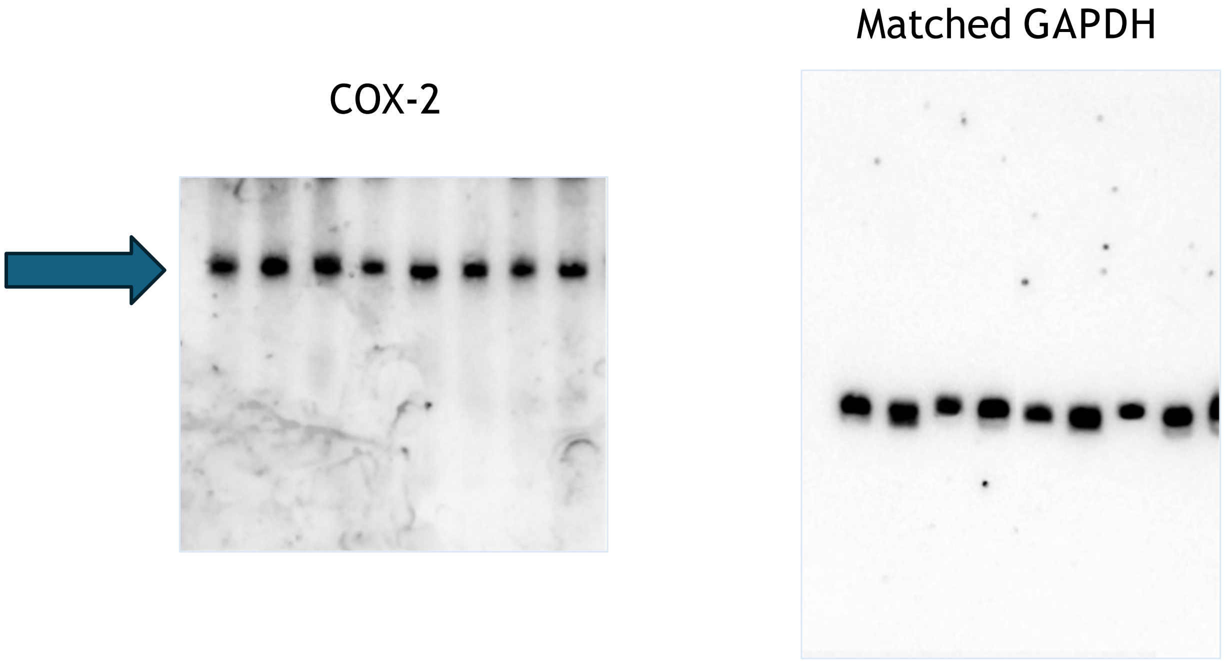

Original uncropped images of western blot analysis shown in Figure S3**:** COX-2 (and matched GAPDH) in the temporal-occipital cortex

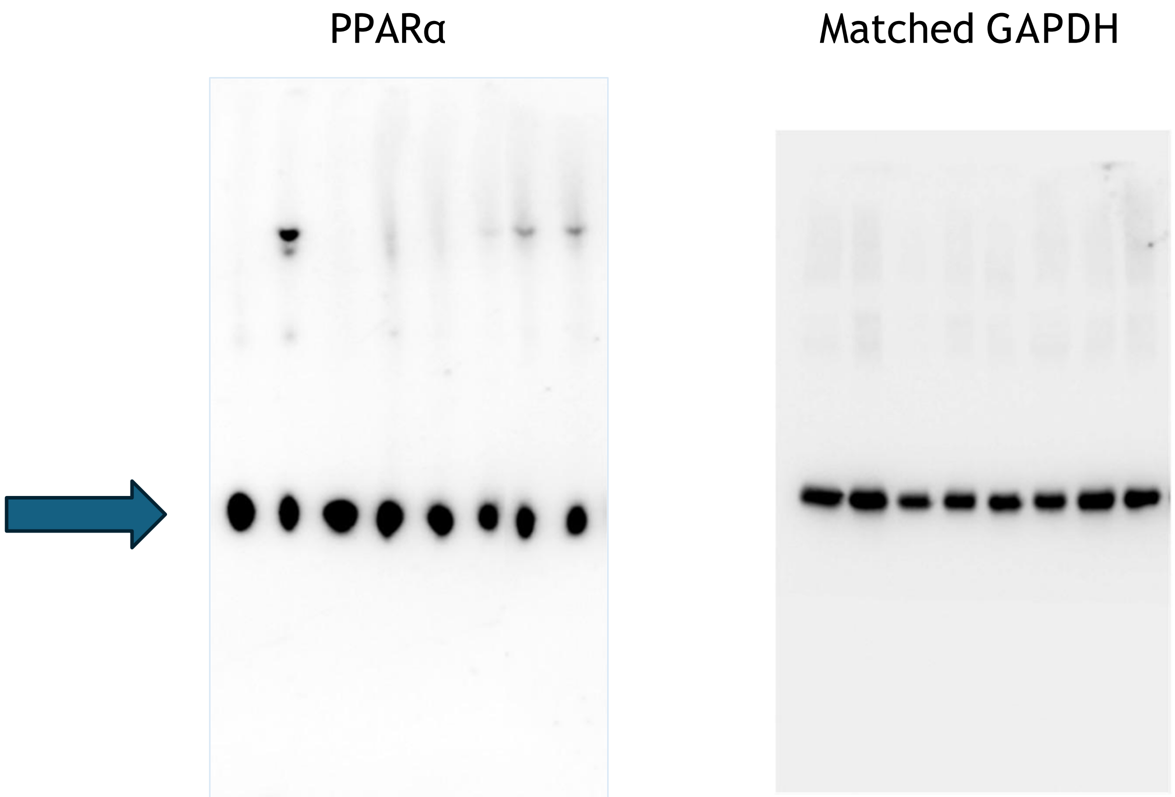

Original uncropped images of western blot analysis shown in Figure S3: **PPARα** (and matched GAPDH) in the temporal-occipital cortex

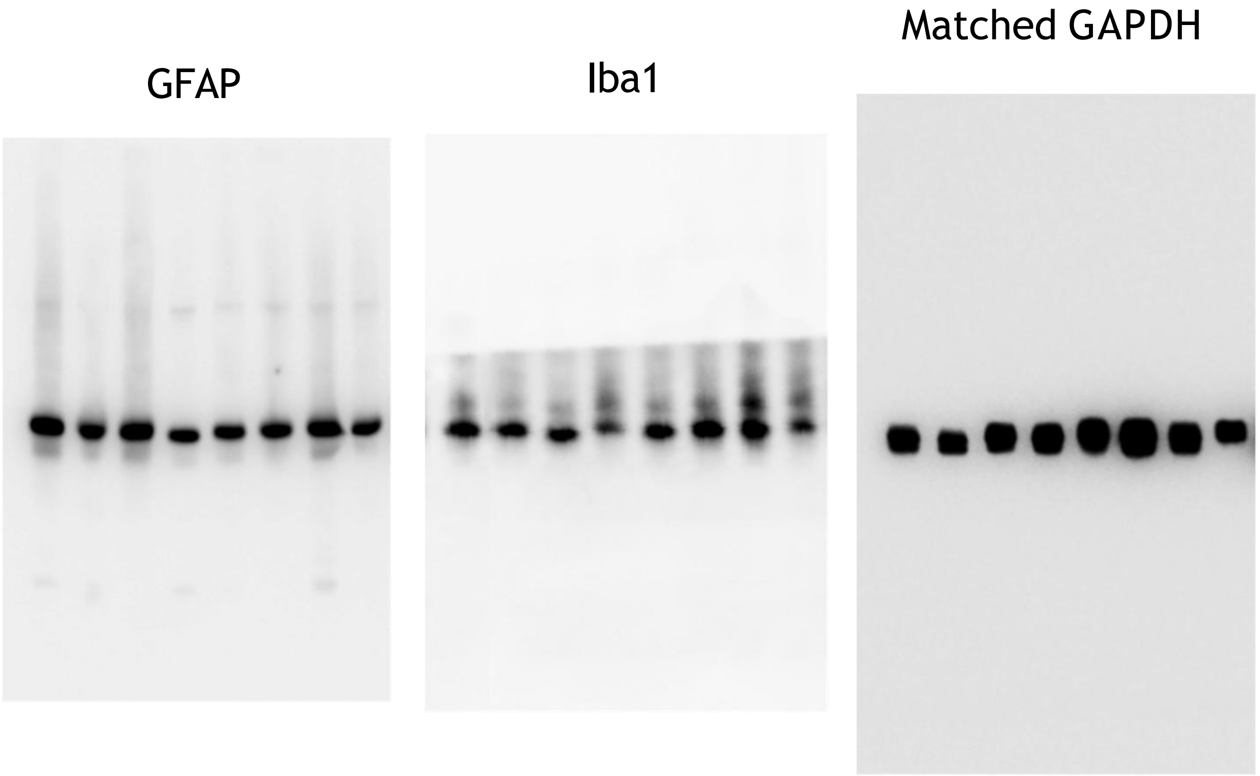

Original uncropped images of western blot analysis shown in Figure S4**:** glial markers GFAP and Iba1 (and matched GAPDH) in the temporal-occipital cortex
