# Supplementary material for: Kaempferol Regulates Lipid Homeostasis, Endocannabinoid System, and PPARα in Rat Cerebral Cortex Following BCCAO/R
Source: Biomolecules. 2025 Oct 11;15(10):1440. doi: 10.3390/biom15101440 (PMC12563104; doi:10.3390/biom15101440)
Supplement: Supplementary file 1 [file biomolecules-15-01440-s001.zip › biomolecules-3839299-SUPPLEM.pdf]

biomolecules

Article

Kaempferol regulates lipid homeostasis, endocannabinoid system, and PPARα in Rat Cerebral Cortex following BCCAO/R

Gianfranca Carta†, Maria Pina Serra†, Elisabetta Murru, Marianna Boi, Claudia Manca, Ylenia Lai, Monica Cabboi, Antonella Carta, Sebastiano Banni*, Marina Quartu*

Department of Biomedical Sciences, University of Cagliari, 09100 Cagliari, Italy; giancarta@unica.it (G.C.); mpserra@unica.it (M.P.S.); memurru@unica.it (E.M.); marianna.boi@unica.it (M.B.); claumanca@unica.it (C.M.); ylenia.lai@unica.it (Y.L.); monica.cabboi3@unica.it (M.C.); antonellacarta@outlook.it (A.C.); banni@unica.it (S.B.); quartu@unica.it (M.Q.)

* Authors to whom correspondence should be addressed.
† These authors contributed equally to this work.

Supplementary Materials: Supplementary materials can be found at www.mdpi.com/xxx/s1.

 https://doi.org/10.3390/xxxxx

Table S1. Statistical data, F-Value and p-Value, performed on eCBs and NAEs concentrations (expressed as mol% of total fatty acid) in the frontal cortex and plasma of the four experimental groups: SHAM-vehicle, SHAM-KAM, bilateral common carotid artery occlusion followed by reperfusion (BCCAO/R)-vehicle and BCCAO/R-kaempferol (KAM) rats.. F-Values and significance levels from two-way ANOVAs performed on data obtained by means of HPLC and HPLC-MS.

	ANOVA factors	BCCAO/R		KAM		BCCAO/R x KAM		
	**Marker**	*F*-Value	*p*-Value	*F*-Value	*p*-Value	*F*-Value	*p*-Value	DF
**Frontal Cortex**	AA_HP	0.734	ns	33.74	< 0.0001	1.828	ns	1,20
	2-AG	6.863	0.0150	32.1	< 0.0001	3.675	ns	1,24
	AEA	1.112	ns	8.464	0.0077	0.8644	ns	1,24
	DHAEA	16.55	0.0004	99.87	< 0.0001	13.00	0.0014	1,24
	PEA	0.0292	ns	35.94	< 0.0001	2.606	ns	1,24
	OEA	0.7689	ns	93.49	< 0.0001	17.45	0.0004	1,22
**Plasma**	AA_HP	0.0686	ns	3.726	ns	5.373	0.0297	1,23
	2-AG	1.934	ns	2.676	ns	3.019	ns	1,24
	AEA	0.6209	ns	20.07	0.0002	0.2235	ns	1,24
	DHAEA	0.2643	ns	90.18	< 0.0001	0.3463	ns	1,22
	PEA	15.36	0.0006	26.80	< 0.0001	9.705	0.0047	1,24
	OEA	11.07	0.0028	13.45	0.0012	20.59	0.0001	1,24

Legend: AA_HP, arachidonic acid hydroperoxides; 2-AG, 2-arachidonoylglycerol; AEA, arachidonoylethanolamide; DHAEA, docosahexaenoylethanolamide; PEA, palmitoylethanolamide; OEA, oleoylethanolamide; DF, degrees of freedom; ns, not statistically significant.

Table S2. Main fatty acid (FA) concentrations in the frontal cortex from the four experimental groups: SHAM-vehicle, SHAM-KAM, bilateral common carotid artery occlusion followed by reperfusion (BCCAO/R)-vehicle and BCCAO/R-kaempferol (KAM) rats. Quantitative data, expressed as mol% of total FA and presented as mean ± SEM (n = 7 per group), were obtained using HPLC and Gas Chromatography.

	**SHAM-vehicle**			**SHAM-KAM**			**BCCAO/R-vehicle**			**BCCAO/R-KAM**		
	Mean		SEM	Mean		SEM	Mean		SEM	Mean		SEM
**12:0**	0.100	±	0.024	0.172	±	0.067	0.169	±	0.096	0.151	±	0.035
**14:0**	0.512	±	0.094	0.518	±	0.065	0.634	±	0.084	0.566	±	0.069
**15:0**	0.197	±	0.035	0.209	±	0.011	0.224	±	0.187	0.156	±	0.039
**16:0**	33.272	±	0.651	33.611	±	0.364	32.979	±	1.115	33.795	±	0.910
**18:0**	20.774	±	0.404	21.313	±	0.424	21.172	±	0.768	21.989	±	0.646
**20:0**	0.236	±	0.048	0.264	±	0.060	0.300	±	0.054	0.255	±	0.066
**22:0**	0.136	±	0.015	0.119	±	0.044	0.112	±	0.064	0.131	±	0.009
**24:0**	0.049	±	0.010	0.061	±	0.016	0.057	±	0.034	0.060	±	0.019
**22:5n3**	0.400	±	0.065	0.403	±	0.129	0.355	±	0.246	0.478	±	0.131
**22:6n3**	11.214	±	0.337	11.075	±	0.412	11.268	±	0.330	10.982	±	0.397
**18:2n6**	1.799	±	0.213	1.366	±	0.077	1.803	±	0.408	1.416	±	0.073
**20:4n6**	11.548	±	0.214	11.457	±	0.456	11.450	±	0.559	11.073	±	0.444
**22:4n6**	2.266	±	0.073	2.239	±	0.035	2.208	±	0.140	2.150	±	0.098
**22:5n6**	1.219	±	0.185	1.191	±	0.103	1.182	±	0.134	1.203	±	0.195
**18:1n9**	15.804	±	0.456	15.454	±	1.163	15.638	±	0.771	15.044	±	0.662
**SFA**	55.276	±	0.661	56.268	±	0.976	55.647	±	1.861	57.102	±	1.242
**UFA**	44.250	±	0.663	43.186	±	0.947	43.903	±	1.895	42.347	±	1.341
**PUFAn3**	11.615	±	0.303	11.478	±	0.356	11.623	±	0.346	11.460	±	0.439
**PUFAn6**	16.832	±	0.217	16.253	±	0.375	16.643	±	0.993	15.842	±	0.531
**PUFAn9**	15.804	±	0.456	15.454	±	1.163	15.638	±	0.771	15.044	±	0.662

Legend: Saturated fatty acids (SFA), polyunsaturated fatty acids (PUFA)

Table S3. Main fatty acid (FA) concentrations in plasma from the four experimental groups: SHAM-vehicle, SHAM-KAM, bilateral common carotid artery occlusion followed by reperfusion (BCCAO/R)-vehicle, and BCCAO/R-kaempferol (KAM) rats. Quantitative data, expressed as mol% of total FA and presented as mean ± SEM (n = 7 per group), were obtained using HPLC and Gas Chromatography.

	**SHAM-vehicle**			**BCCAO/R-vehicle**			**SHAM-KAM**			**BCCAO/R-KAM**		
	Mean		SEM	Mean		SEM	Mean		SEM	Mean		SEM
**14:0**	0.895	±	0.312	0.714	±	0.078	0.700	±	0.194	0.755	±	0.080
**15:0**	0.551	±	0.263	0.453	±	0.054	0.481	±	0.134	0.522	±	0.201
**16:0**	24.697	±	1.013	22.777	±	0.605	23.180	±	1.279	25.514	±	2.158
**18:0**	11.356	±	0.170	11.798	±	0.831	10.675	±	0.970	11.561	±	2.024
**20:0**	0.248	±	0.060	0.203	±	0.040	0.156	±	0.017	0.160	±	0.054
**22:0**	0.187	±	0.080	0.163	±	0.043	0.118	±	0.034	0.149	±	0.063
**24:0**	0.531	±	0.167	0.512	±	0.095	0.662	±	0.194	0.453	±	0.114
**18:3n3**	0.671	±	0.065	0.561	±	0.161	0.928	±	0.488	0.793	±	0.134
**20:5n3**	0.307	±	0.031	0.261	±	0.102	0.358	±	0.164	0.384	±	0.075
**22:5n3**	0.703	±	0.130	0.751	±	0.126	0.689	±	0.095	0.823	±	0.119
**22:6n3**	2.444	±	0.442	2.658	±	0.103	2.697	±	0.617	2.394	±	0.316
**16:2n6**	0.080	±	0.010	0.075	±	0.017	0.085	±	0.043	0.085	±	0.015
**18:2n6**	19.992	±	1.169	19.577	±	2.914	27.905	±	9.305	22.317	±	2.360
**18:3n6**	0.385	±	0.108	0.403	±	0.094	0.394	±	0.186	0.363	±	0.053
**20:2n6**	0.419	±	0.192	0.497	±	0.078	0.469	±	0.145	0.342	±	0.044
**20:3n6**	0.345	±	0.069	0.358	±	0.017	0.311	±	0.081	0.250	±	0.062
**22:4n6**	0.278	±	0.058	0.280	±	0.018	0.382	±	0.110	0.303	±	0.012
**18:1n9**	9.283	±	0.452	7.766	±	0.626	11.201	±	4.448	9.495	±	1.282
**20:3n9**	0.071	±	0.022	0.084	±	0.032	0.103	±	0.035	0.066	±	0.008
**SFA**	38.465	±	1.048	36.619	±	1.429	26.979	±	18.113	39.115	±	3.718
**UFA**	56.130	±	1.738	56.681	±	2.633	67.507	±	17.611	56.787	±	3.641
**PUFAn3**	4.125	±	0.404	4.231	±	0.327	4.500	±	0.907	4.393	±	0.394
**PUFAn6**	42.402	±	1.556	44.314	±	2.033	51.514	±	12.223	42.682	±	2.715
**PUFAn9**	9.354	±	0.464	7.850	±	0.627	11.304	±	4.480	9.562	±	1.277

Legend: Saturated fatty acids (SFA), polyunsaturated fatty acids (PUFA)

Ta**ble S4.** Statistical data, F-Value and p-Value, performed on relative protein levels of CB1R, CB2R, COX-2, PPAR〈, and glial markers GFAP and Iba1 obtained in homogenates of frontal cortex and temporal-occipital cortex from the four experimental groups: SHAM-vehicle, SHAM-KAM, bilateral common carotid artery occlusion followed by reperfusion (BCCAO/R)-vehicle and BCCAO/R-kaempferol (KAM) rats. *F*-Values and significance levels from two-way ANOVAs performed on data obtained using Western Blot.

	**ANOVA factors**	BCCAO/R		KAM		BCCAO/R x KAM		
	**Marker**	*F*-Value	*p*-Value	*F*-Value	*p*-Value	*F*-Value	*p*-Value	DF
**Frontal cortex**	CB1R	9.280	0.005	50.86	< 0.0001	2.945	n.s	1,28
	CB2R	2.187	n.s.	42.54	< 0.0001	0.2870	n.s	1,28
	COX-2	2.531	n.s.	15.36	0.0005	0.0010	n.s.	1,28
	PPARα	27.52	< 0.0001	52.78	< 0.0001	9.023	0.0056	1,28
	GFAP	8.405	0.0072	16.90	0.0003	4.683	0.0391	1,28
	Iba1	2.316	n.s.	21.97	<0.0001	0.5667	n.s.	1,28
**Temporal-occipital cortex**	CB1R	0.22	n.s	4.09	n.s.	2.7	n.s.	1,28
	CB2R	4.164	n.s.	0.066	n.s.	0.221	n.s.	1,28
	COX-2	0.21	n.s.	0.29	n.s.	1.123	n.s.	1,28
	PPARα	0.07	n.s.	3.64	n.s.	0.931	n.s	1,28
	GFAP	0.009	n.s.	0.148	n.s.	0.39	n.s.	1,28
	Iba1	2.79	n.s.	0.25	n.s.	0.09	n.s.	1,28

Legend: DF, degrees of freedom; ns, not statistically significant

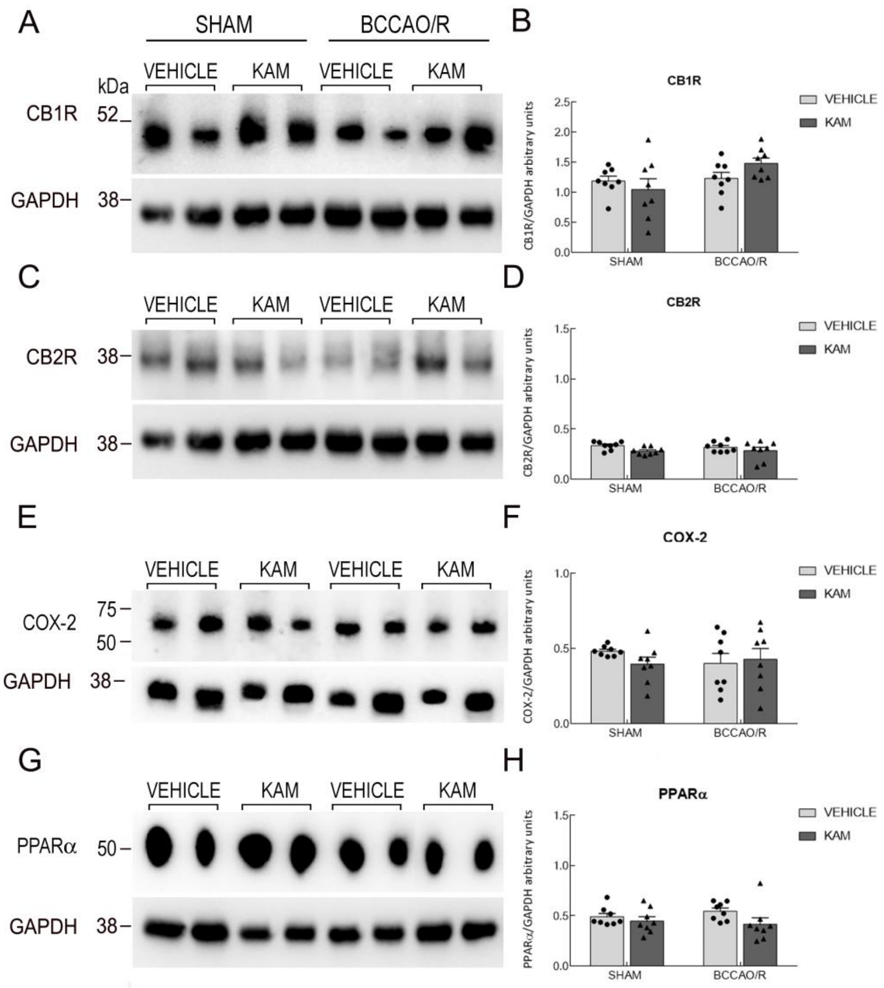

Figure S1. Western blot analysis was conducted to assess cannabinoid receptors CB1R (**A**,**B**) and CB2R (**C**,**D**), cyclooxygenase-2 (COX-2) (**E**,**F**), and peroxisome-proliferator activated receptor α (PPARα) (**G**,**H**) in the temporal-occipital cortex of four experimental groups: SHAM-vehicle, SHAM- kaempferol (KAM), bilateral common carotid artery occlusion followed by reperfusion (BCCAO/R)-vehicle and BCCAO/R-KAM rats. (**B**,**D**,**F**,**H**) Densitometric analysis of the band gray levels, expressed as a percentage of the optical density (O.D.) ratio, was performed for immunostained bands of CB1R, CB2R, COX-2, and PPARα relative to GAPDH. Graphs represent mean values (bars) and superimposed individual data points for vehicle-treated (n = 8 for both SHAM-vehicle and BCCAO/R-vehicle) (•) and KAM-treated (n = 8 for both SHAM-KAM and BCCAO/R-KAM) (▲) rats. Error bars represent the standard error of the mean (S.E.M.). Statistical differences between groups were evaluated using two-way ANOVA. Refer to Table 1S for F- and p-values related to the effects of BCCAO/R, KAM pre-treatment, and their interaction.

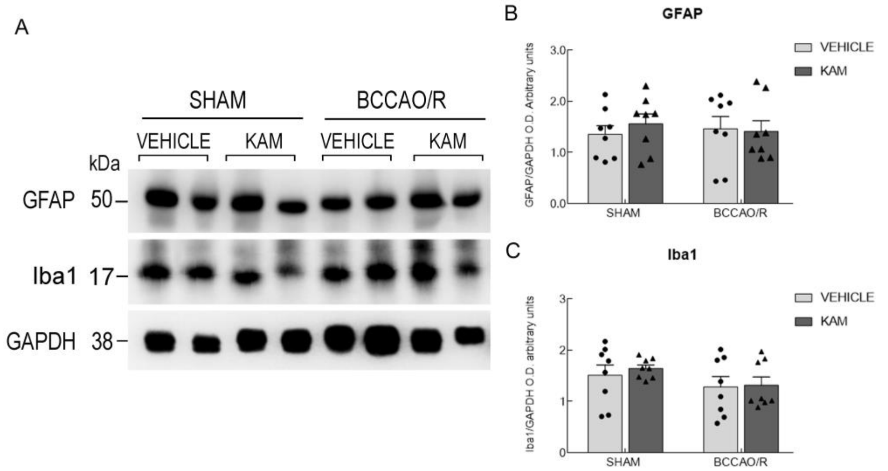

Figure S2. Western blot analysis (**A**) of glial fibrillary acidic protein (GFAP) and ionized calcium-binding adaptor molecule 1 (Iba1) in the temporal-occipital cortex of sham-operated and rats subjected to bilateral common carotid artery occlusion followed by reperfusion (BCCAO/R). Both vehicle-treated and kaempferol (KAM) pre-treated groups are shown. (**B, C**) Densitometric analysis, with band gray levels expressed as the percentage of the optical density (O.D.) ratio of GFAP- and Iba1-immunostained bands to GAPDH. Graphs represent mean values (bars) and superimposed individual data points for vehicle-treated (n = 8 for both SHAM-vehicle and BCCAO/R-vehicle) (•) and KAM-treated (n = 8 for both SHAM-KAM and BCCAO/R-KAM) (▲) rats. Error bars indicate the standard error of the mean (S.E.M.). Statistical differences between groups were evaluated using two-way ANOVA. Refer to Table 1S for F- and p-values related to the effects of BCCAO/R, KAM pre-treatment, and their interaction.

Disclaimer/Publisher's Note: The statements, opinions and data contained in all publications are solely those of the individual author(s) and contributor(s) and not of MDPI and/or the editor(s). MDPI and/or the editor(s) disclaim responsibility for any injury to people or property resulting from any ideas, methods, instructions or products referred to in the content.
